# Polyelectrolyte Design Principles for Electrophoretic Drug Delivery

**DOI:** 10.1002/advs.202522981

**Published:** 2026-03-15

**Authors:** Helena Saarela Unemo, Iwona Bernacka‐Wojcik, Lingkai Zhu, Marle E. J. Vleugels, Moa E. Hörberg, Caroline Lindholm, Magnus Berggren, Daniel T. Simon, Theresia Arbring Sjöström

**Affiliations:** ^1^ Laboratory of Organic Electronics Department of Science and Technology Linköping University Norrköping Sweden; ^2^ Wallenberg Initiative Materials Science for Sustainability Department of Science and Technology Linköping University Norrköping Sweden; ^3^ Department of Biomedical Engineering Linköping University Linköping Sweden

**Keywords:** drug delivery, electrophoretic transport, ion‐conducting polymers, iontronics, structure–property–function relationships

## Abstract

Soft and tunable materials that facilitate electroactive control over molecular mass transport are key to advancing bioelectronic and therapeutic technologies. Iontronic drug delivery devices rely on polyelectrolytes that act as solid‐state ionic conductors, where drug transport and on‐demand release are controlled by applied electric potentials. Achieving precise dosing requires polyelectrolytes that selectively transport drug‐scale molecules with high conductivity. For drug‐sized molecules > 200 g mol^−^^1^, achieving these traits simultaneously remains a central challenge that calls for targeted material optimization. Here, we report the design space of polyelectrolytes to improve performance. We systematically varied the composition of AMPS:PEGDA polyelectrolytes and mapped the structure–property–function relationships using a drug‐sized model molecule (cytidine, 243 g mol^−1^) in relevant device architecture for implantable drug delivery systems. A multiparameter design map identifies quantitative design rules: pair high hydration to sustain transport with balanced fixed charge density to keep loading dynamics manageable, without sacrificing selectivity. Small‐angle X‐ray scattering reveals that nanoscale domain spacing and short‐range order correlate directly with conductivity and efficiency. Optimized formulations outperform previous generations by achieving near‐theoretical delivery efficiencies with minimal sacrifice of ionic conductivity.

## Graphical abstract

Structure–property–function mapping of polyelectrolytes reveals how composition and nanoscale order control on‐demand electrophoretic drug transport. Charge density quantified under implant‐relevant conditions shows that encapsulation limits swelling, enabling high effective charge density. A design map defines a water‐ and charge‐rich, tunable window where selectivity and mobility coexist, guiding future design of devices for programmable high‐precision dosing.

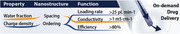

## 1 Introduction

Achieving precise control over drug exposure in space and time, at efficacious yet safe concentrations, remains a central challenge across medicine. Systemic drug delivery must overcome biological barriers and tolerability constraints, which often lead to subtherapeutic levels at the target and/or off‐target exposure elsewhere in the body. Implantable drug‐delivery systems (IDDS) address this by enabling localized, temporally programmed dosing while limiting systemic burden [1]. Achieving on‐demand and programmable dosing is mostly implemented in fluidic‐based devices, where pressure delivers drug solutions into tissue targets. Surveys show that ∼80%–95% of marketed drugs contain ionizable groups, and more than 60% of those are charged at physiological pH [2, 3]. This allows for an alternative strategy, where drug delivery can be facilitated and controlled electrically, with drugs migrating under an applied field in solid‐state matrices, without transport of bulk fluid and without adding unnecessary pressure gradients in sensitive biological targets.

Soft materials, such as biocompatible hydrogels, are particularly attractive for such minimally invasive, yet highly controlled, drug delivery strategies, because of their tissue‐like mechanics, high water content, and tunable physical and chemical properties [4]. Charged hydrogels, i.e., polyelectrolytes, enable selective loading and transport of anionic or cationic therapeutic species in the solid‐state matrix. Polyelectrolytes integrated into device‐relevant geometries connected to a source reservoir form a drug delivery platform where loading, transport, and delivery can be actively modulated by external potentials. This mode of operation, referred to as electrophoretic or iontronic drug delivery [5], has been used by our group and others to deliver a variety of charged bioactive compounds [6, 7, 8, 9, 10, 11, 12, 13, 14, 15, 16]. Previous studies have demonstrated electrophoretic delivery of a wide range of charged small‐molecule compounds (i.e., 39–525 g mol^−1^) using various iontronic platforms. These include potassium ions [6, 8], neurotransmitters such as acetylcholine [7] and γ‐aminobutyric acid (GABA) [9, 10], plant hormones such as abscisic acid [11], local anesthetics such as bupivacaine [12], and chemotherapeutics such as gemcitabine [13, 17] and doxorubicin [14]. However, transport efficiency (the fraction of charge carried by the drug) remains far below theoretical limits and shows considerable variability, especially for drug‐sized ions (> 200 g mol^−1^). For example, reported efficiencies include 48% for gemcitabine [17] and 26% for indigo carmine [16], whereas ions such as fluoxetine [15] and doxorubicin [14] are delivered at efficiencies below 4%. This low predictability in dosing represents challenges that must be addressed before clinical translation of electrophoretic drug delivery devices.

**FIGURE 1:**
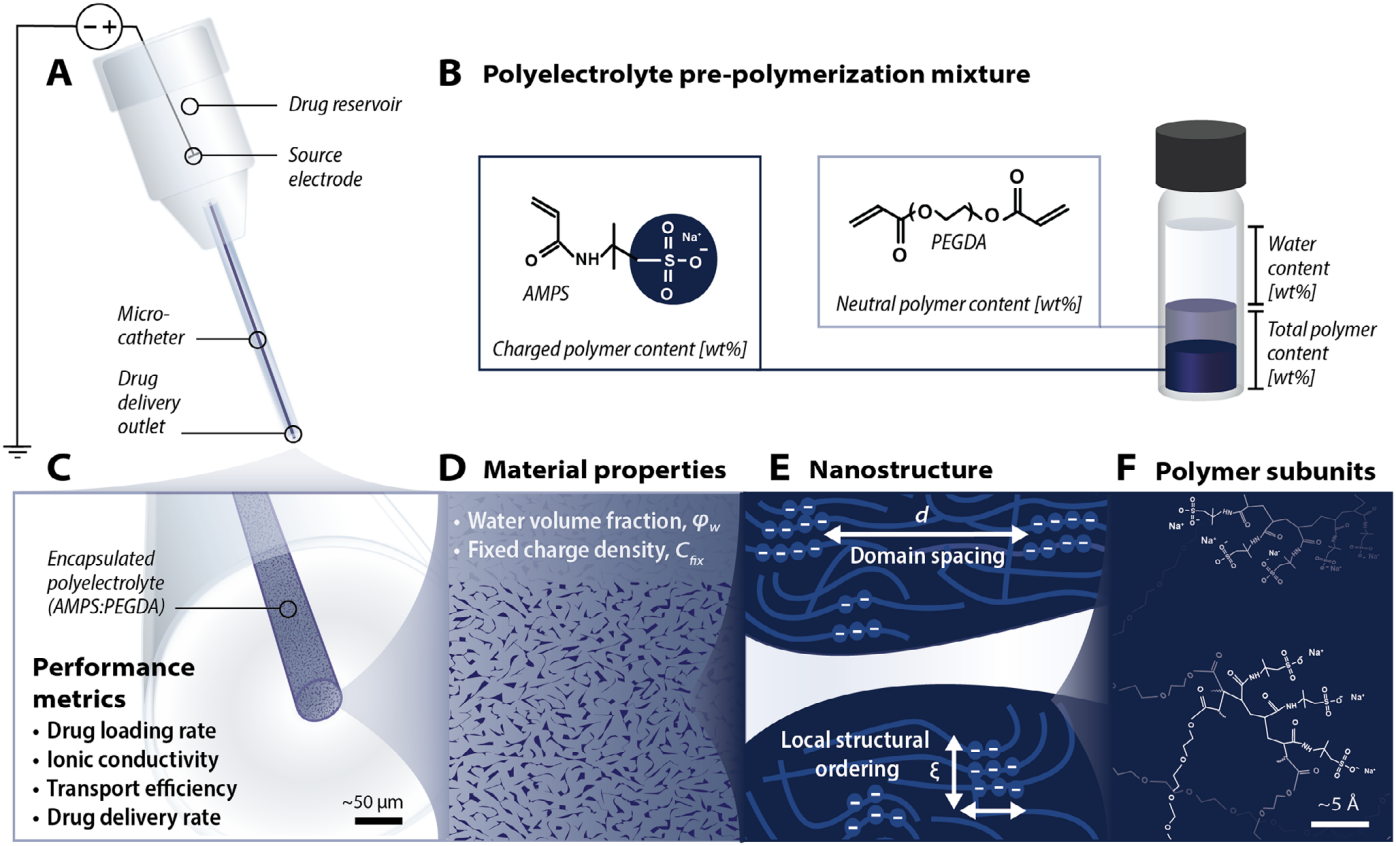
Multiscale view of an electrophoretic drug‐delivery device, with the design space and structure–property–function landscape investigated in this work. (A) The device concept for electrophoretic drug delivery consists of micro‐catheters (polyimide‐coated glass capillaries filled with an anionic polyelectrolyte) separating the drug reservoir and the target medium. An applied potential between the source electrode and ground, selectively drives ionic (here cationic) drugs from the reservoir toward the device outlet. (B) A polymer fraction containing a charged monomer; AMPS (2‐acrylamido‐2‐methyl‐1‐propanesulfonic acid), and a neutral polymer; PEGDA (polyethylene glycol diacrylate), together with a water fraction, forms a pre‐polymerization composition mixture. (C) Zoom at the device outlet illustrates encapsulated polyelectrolyte network. The main device‐level performance metrics include drug loading rate (in pL min^−1^) and drug delivery rate (to the target, in pmol min^−1^), further governed by key material performance metrics: ionic conductivity (σ) and efficiency (fraction of charge carried by the drug). (D) Characteristic random phase separations between water‐ and polymer‐rich phases in the polyelectrolyte network. Key macro‐scale material properties are water volume fraction (Φ_w_) and fixed charge density (*C*_fix_). (E) Nanostructural characteristics represented by domain spacing (d) and local structural ordering (ξ), emerge from polymer/water fractions and charge distribution. (F) Polymeric subunits form random cross‐links that shape the polymerized polyelectrolyte network AMPS:PEGDA.

In this study, we aim to improve the transport performance of polyelectrolytes for electrophoretic drug delivery systems. Our approach builds on optimization principles established for ion exchange membranes in energy and separation technologies [18, 19, 20, 21]. For transport of drug sized organic ions in implant‐relevant device geometries, these links are less established. Here, we translate established membrane design logic to polyelectrolyte micro‐catheters and present a systematic investigation of the structure–property–function relationships that link composition, nanoscale structure, and device function (Figure 1), to identify and formulate practical optimization guidelines for the use‐case electrophoretic drug delivery. Using AMPS (2‐acrylamido‐2‐methyl‐1‐propanesulfonic acid) and PEGDA (polyethylene glycol diacrylate), AMPS:PEGDA, we vary charge, polymer, and water fractions in the pre‐polymerization mixture, to explore a compositional matrix (Figure 1B). To ensure relevance for implantable systems, this investigation was mainly performed on polyelectrolyte micro‐catheters, i.e., polyelectrolytes encapsulated in coated glass capillaries (Figure 1C). At the device level, performance is summarized by two key metrics: drug loading rate (describing volumetric uptake of drug ions; pL min^−1^) and drug delivery rate (molar flux of drug released; pmol min^−1^). The corresponding material‐level metrics: ionic conductivity (*σ*) and transport efficiency, are known to be tightly linked to the balance between polymer, charged units, and water [18, 20]. This balance can be quantified by two key macro‐scale material properties of the polyelectrolyte network; the water volume fraction (*Φ*_w_), and fixed charge density (*C*_fix_) (Figure 1D) [20]. *Φ*_w_ is defined as the ratio of water volume to the total hydrated polymer volume, and is commonly used as a proxy for mesh size because it governs molecular diffusion. *C*_fix_ is defined as the molar concentration of immobile charges per hydrated volume. *C*_fix_ is a key parameter governing charge selectivity and ionic conductivity, and is recognized as a figure of merit for polyelectrolyte optimization, in particular for ions of molecular weight < 100 g mol^−1^ [20, 21]. Furthermore, the polymer architecture is shaped by characteristic nanostructural organization (Figure 1E), such as domain spacing (*d*), and order parameter (*ξ*), typically characterized using small‐angle X‐ray scattering (SAXS) [22, 23]. A smaller *d* indicates more closely spaced ionic clusters, while a larger *ξ* suggests more pronounced short‐range order or connectivity among the hydrophilic domains [24, 25, 26]. The balance between the polymeric subunits (Figure 1F) is limited by practical design trade‐offs. AMPS introduces fixed charges, while PEGDA primarily controls crosslink density to prevent excessive swelling and preserve mechanical integrity. Water provides spacing for molecular mobility within the network, while very high *Φ*_w_ will dilute *C*_fix_ and reduce selectivity. To probe transport and delivery of drug‐sized molecules, we used cytidine (243 g mol^−1^), a non‐toxic nucleoside scaffold with several FDA‐approved derivatives in oncology and antiviral therapy [27]. Our aim is not to claim universality, but to quantify how composition and confinement set *Φ*_w_, *C*_fix_, and nanostructure, and how these jointly control loading, conductivity, and delivery for one representative material‐ion pair under implant‐relevant confinement. As ion chemistry or polyelectrolyte network chemistry changes, the same framework can be repeated to derive tuned design guidelines.

## 2 Results and Discussions

We fabricated polyelectrolyte micro‐catheters by filling polyimide coated glass capillaries (50/375 µm ID/OD) with AMPS:PEGDA polyelectrolyte with varying compositions of pre‐polymerization mixtures. Specifically, we varied the mass fraction of polymer to water (defined as polymer content, wt.%) and the mass fraction of AMPS with respect to the total polymer content (defined as charged polymer content, wt.%). All polyelectrolyte compositions used in this study can be found in Table S1. Device performance metrics, cytidine drug loading rate, delivery rate, and efficiency, and ionic conductivity, were quantified using the standard two‐electrode configuration depicted in Figure 1A (see also Figure S1). Across the studied formulation matrix, sodium transport was quantified for all compositions, whereas cytidine transport and SAXS‐based structural analysis were restricted to subsets due to voltage limit and polyelectrolyte swelling (see Methods for inclusion criteria and dataset sizes).

### 2.1 Key Function‐Structure‐Property Relationships

**FIGURE 2:**
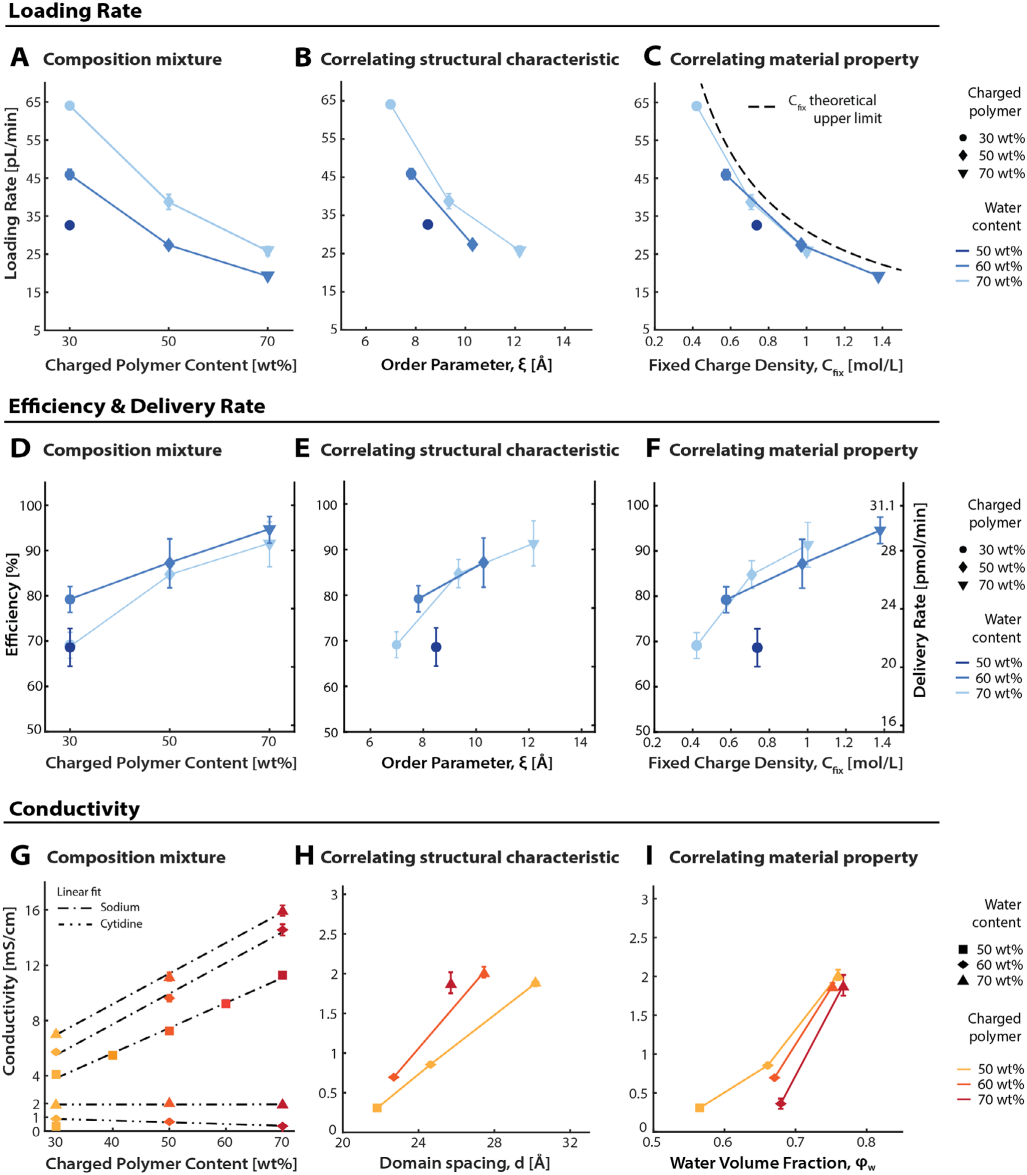
Relationships between polyelectrolyte structure, charge characteristics, and functional drug delivery performance. **Row 1: Drug loading dynamics**. (A) Loading rate (pL min^−1^) vs. charged polymer content. 7 compositions; *n* = 6–8 independent replicates per composition (after outlier exclusion). (B) Loading rate vs. structural order parameter ξ. 5 compositions; *n* = 6–8. (C) Loading rate vs. *C*_fix_. Line indicates theoretical upper limit under ideal ion exchange. 7 compositions; *n* = 6–8. **Row 2: Delivery performance**. (D) Efficiency of cytidine delivery vs. charged polymer content. 7 compositions; *n* = 7–8. (E) Cytidine delivery vs. structural order parameter ξ. 5 compositions; *n* = 7–8. (F) Cytidine delivery vs. *C*_fix_. At a driving current of 50 nA, a delivery rate of 31.1 pmol min^−1^ equals 100% efficiency. 7 compositions; *n* = 7–8. **Row 3: Ionic conductivity**. (G) Ionic conductivity (Na^+^ and cytidine) vs. charged polymer content. Dashed lines represent linear regression, and different responses to charged content, for the two different charge carriers. Na^+^ conductivity: 11 compositions; *n* = 6–8. Cytidine conductivity: 7 compositions; *n* = 7–8. (H) Cytidine conductivity vs. domain spacing d. 5 compositions; *n* = 7–8. (I) Cytidine conductivity vs. water volume fraction Φ_w_. 7 compositions; *n* = 7–8. All solid lines in colors connecting the data points are added to guide the eye across the data trend. Note the different color gradients in panel (A–F), compared to panel (G–I).

The key device and material performance metrics examined, along with their dependence on composition, can be seen in the first column of Figure 2. The second and third columns show the nanostructural feature and the material's property with the highest correlation to that device's functionality. A Spearman correlation matrix can be found in Figure S2. The first row of Figure 2 (Figure 2A–C) shows loading dynamics, which reflect ion‐exchange kinetics between Na^+^ (native counter‐ion in our AMPS:PEGDA hydrogels) and cytidine under an applied electric field. Specifically, the drug loading rate reports how quickly resident Na^+^ are replaced by cytidine under a constant current of 50 nA. Higher charged polymer content and lower water content are associated with lower loading rates (Figure 2A). A similar trend is observed with *C*_fix_ (Figure 2C): a larger fixed charge density requires exchange of more Na^+^ ions, slowing the loading process and thereby reducing the loading rate. This trend is supported by a strong negative correlation between loading rate and *C*_fix_ (ρ = –0.984, p = 1.98E‐37). The practical difference on the device scale (catheters of approx. 10 mm in length) results in a time for full exchange between Na^+^ and cytidine and stabilization ranging from 5 h for the devices with the fastest loading to 17 h for the slowest loading (see Figures S3–S9). The dashed line in Figure 2C indicates the theoretical upper bound for loading rate under ideal exchange conditions for the corresponding *C*_fix_ range [16, 28, 29]. The differences between experimental data and this theoretical upper limit are discussed later.

We next investigated delivery performance. Devices delivered cytidine into fresh target electrolyte under a constant 50 nA current for 24 h, and cytidine concentration was quantified by monitoring the optical absorbance at 271 nm. Transport efficiencies, expressed as the fraction of total charge transported through the device carried by the ionic drug, were calculated as described in the experimental section, by comparing the applied current (50 nA, corresponding to a theoretical maximum of 31.1 pmol min^−1^) to the experimentally quantified cytidine in the target electrolyte (Figure 2F). For intuition, 30 pmol min^−^^1^ corresponds to approximately 10–30 µg day^−^^1^ for small molecule drugs in the 200–600 g mol^−^^1^ range. As seen in Figure 2D–F, highest transport efficiency is linked to highest charged polymer content (ρ = 0.882, p = 1.33E‐18), and, at a fixed polymer content, linked to higher structural order (*ξ;* ρ = 0.800, p = 2.52E‐11) and higher *C*_fix_ (ρ = 0.759, p = 2.81E‐11). These trends agree with classic transport theory, which predicts that more charged sites help to maintain a higher selectivity of the ionic drug by limiting inclusion and backflow of small competing co‐ions such as Cl^−^ [18, 20]. Lower *C*_fix_ allows for greater inclusion of co‐ions under constant‐current operation, which lowers selectivity and transport efficiency. Interestingly, however, the transport efficiencies remained relatively high across all formulations (Figure 2D). Notably, these polyelectrolyte formulations enabled significantly higher delivery efficiency (up to 96 ± 1%) than previously reported polyelectrolyte systems delivering drug‐sized ions (4%–48%) [13, 14, 15, 16, 17], including our reported AMPS:PEGDA mixtures delivering the cytidine‐analog gemcitabine (16% efficiency at 20 nA) [13]. The latest AMPS:PEGDA systems featured substantially lower fraction of PEGDA (95 wt.% AMPS to 5 wt.% PEGDA), in combination with low water content (49 wt.%) compared to the present series, which span AMPS fractions of 30–70 wt.% of the polymer fraction, and water contents of 50–75 wt.%.

Ionic conductivity (Figure 2G–I) was obtained by measuring the steady‐state voltage for each ion by applying a series of low currents, up to 100 nA (see Figures S3–S14). We first observed that Na^+^ conductivity increased with charged polymer content (ρ = 0.876, p = 1.24E‐28) and *C*_fix_, and as well as with hydration (*Φ*_w_; ρ = 0.707, p = 2.02E‐14) within our parametric range. These results are consistent with classical transport theory, where small, highly mobile counter‐ions dominate conduction when many charged sites are available [18, 20]. For our therapeutic model molecule cytidine, however, the domain spacing (Figure 2H) and *Φ*_w_ (Figure 2I) seemingly dictate conductivity, both supported by strong positive Spearman correlations, ρ = 0.917 (p = 2.58E‐18) and ρ = 0.728 (p = 9.71E‐10), respectively. No statistically significant correlation was observed between charge content and cytidine conductivity (ρ = 0.055, p = 0.700). This indicates that, within this parametric space, cytidine mobility is primarily constrained by a tighter network nanostructure (smaller *d*) rather than by a shortage of available charge carrier sites. In other words, increasing charge content does raise Na^+^ conductivity but does not raise cytidine conductivity in this window, because the larger ion is more strongly hindered by a tighter nanostructure than small counter ions.

Overall, we found that each performance metric is mainly dominated by one specific aspect of the polyelectrolyte composition, material property and nanostructural characteristic. Loading rate scales inversely with *C*_fix_, while efficiency increases with increasing *C*_fix_. In practice, this means that small improvements in transport efficiency can lead to substantial increases in device resistance (Figure S15) and prolonged loading times. Consequently, higher operating voltages are required even at low driving currents, and switching between on‐ and off‐states becomes slower in response. In contrast to the charge‐dependent scaling of sodium conductivity, we see that the conductivity of cytidine in this set of material formulations is governed primarily by water content. Thus, these observations hint at the classic permeability‐selectivity trade‐off in polyelectrolytes, but also suggest that careful tuning of *C*_fix_ allows for high efficiency to be retained, while increasing *Φ*_w_ optimizes for drug loading and conductivity. We therefore proceed with an in‐depth analysis of *C*_fix_ and *Φ*_w_, to understand how they differ with respect to polymer composition and physical constraints in implantable devices, and how interconnections between these parameters affect drug delivery.

### 2.2 Polyelectrolyte Composition and Encapsulation Control Hydration and Effective Charge Density

**FIGURE 3:**
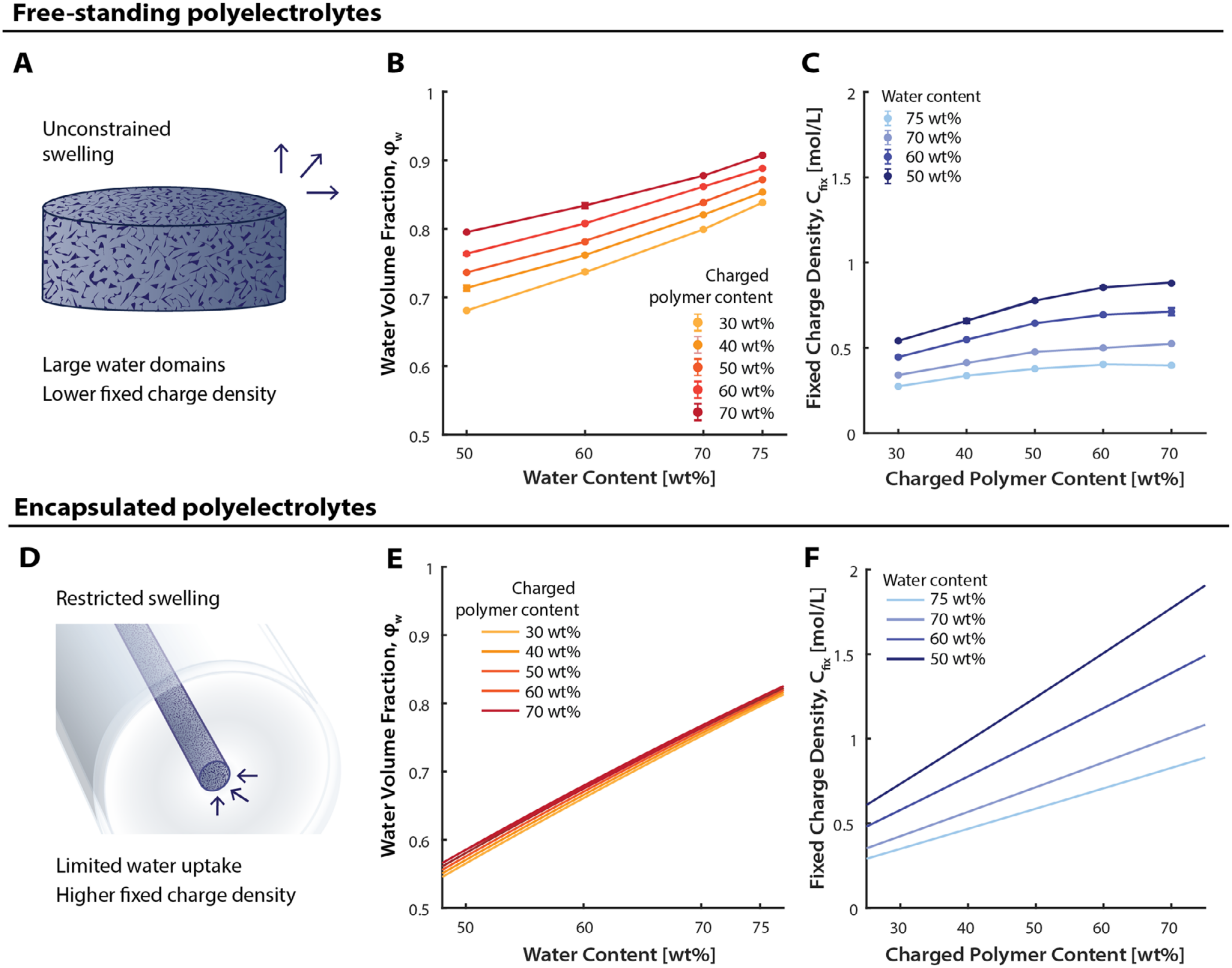
Hydration and fixed charge characteristics of AMPS:PEGDA polyelectrolytes as a function of composition, in free‐swelling discs (A–C) vs. encapsulated form (D–F). (A) Schematic illustration of unconstrained swelling in free‐standing polyelectrolytes. (B) Maximum Φ_w_ after swelling in deionized water, as a function of water content in the polymer composition matrix. Color gradient indicates shift in charged polymer content (AMPS:PEGDA ratio). (C) *C*_fix_ as a function of charged polymer content for free‐swelling conditions. In panel (B,C), lines are added to guide the eye across experimental trends. Water volume fraction and fixed charge density are shown as mean ± SD (*n* = 3) for 20 free‐standing compositions; error bars are barely visible. (D) Schematic illustration of encapsulated polyelectrolytes, where physical constraints restrict swelling. (E) Analytical estimation of effective Φ_w_ based on material composition and geometrical constraints, as a function of water content in the polymer composition matrix. (F) Estimated *C*_fix_ in encapsulated polyelectrolytes, as a function of charged polymer content in the polymer composition matrix.

To investigate the mechanisms underlying the improved performance of the AMPS:PEGDA devices, we systematically characterized how these dictating parameters, i.e., *Φ*_w_ and *C*_fix_, themselves are set by polymer composition and physical constraints. These polyelectrolyte characteristics are conventionally studied in the free‐standing disc form (Figure 3A), where swelling can be assessed directly by measuring water uptake when soaked in deionized water [30, 31, 32]. We performed such a characterization for a broad range of material compositions (Table S1). As seen in Figure 3B, water uptake, and the associated *Φ*_w_, were found to increase with increasing water content and higher charged polymer content in the pre‐polymerization mixture. Increasing the charged polymer fraction from 30 to 50 wt.% of the total polymer fraction leads to higher *C*_fix_ (Figure 3C), while beyond 60 wt.% the *C*_fix_ reaches a plateau. These trends, including the plateau, are consistent with previous reports. This plateau is attributed to excessive swelling at high ionic content, which dilutes the fixed charges and limits further increases in *C*_fix_ [30, 31, 32].

Encapsulated hydrogels, as shown in Figure 3D, are subject to additional physical constraints on swelling beyond those imposed by crosslinking [33]. We therefore hypothesize that conventional methods developed for free‐standing materials cannot directly be applied to our system, as we encapsulate the polyelectrolytes in micro‐catheters. Therefore, we here estimated *C*_fix_ for encapsulated polyelectrolytes by accounting for the polyelectrolyte composition mixture together with geometric restrictions in the microcatheter. Specifically, we assume that the swelling of the polyelectrolyte in the glass capillary is negligible, meaning that the *Φ*_w_ of encapsulated devices corresponds to the water volume added to the pre‐polymerization mixture. The *Φ*_w_ were further refined using polymer densities extrapolated from Yan et al. [31] (Figure S16). Since this method does not allow for any swelling beyond the geometrical constraints, the derived values should be interpreted as effective *Φ*_w_ and effective *C*_fix_, rather than the maximum *Φ*_w_ and corresponding *C*_fix_ derived in the free‐standing case. The results from this estimation can be seen in Figure 3E,F. The effective *Φ*_w_ increased linearly with water content, with a slight offset due to differences in polymer density. In contrast to the free‐standing case, *C*_fix_ under constrained conditions rose linearly across the full range of increasing charged polymer content (Figure 3F), reaching higher overall *C*_fix_. This estimation assumes ideal polymerization (i.e., negligible loss of un‐crosslinked monomers during soaking), leading to *C*_fix_ values that may slightly overestimate the true value. Outside our tested range, particularly at very low water content or very high charged polymer fractions, we expect predictions to deviate further due to excessive density or insufficient crosslinking (see Supporting Information and Figure S17 for further reasoning). Note that the tested compositions in this study do not approach extreme limits where the *C*_fix_ estimation becomes invalid.

For comparison, *C*_fix_ of encapsulated polyelectrolytes for electrophoretic drug delivery devices has been estimated in previous studies during active drug loading [16, 28, 29]. Specifically, *C*_fix_ was determined from the total charge transferred (integrated current) during gradual ion exchange after switching the source reservoir from high‐ to low‐mobility counterions, which thereby replaced the initially bound ions within the polyelectrolyte network. This approach assumes purely active ion exchange (i.e., no passive exchange) and no unselective transport during the ion exchange and should be interpreted as an upper‐bound estimate for *C*_fix_ for the recorded loading rate, as indicated in Figure 2C. We note that this ion‐exchange‐based estimation agrees reasonably well with our experimental loading rate data (Figure 2C), yet estimates a higher *C*_fix_ compared to our composition‐based estimation. Taken together, both the ion‐exchange‐based approach and our composition‐based estimation provide upper‐limit values of *C*_fix_ under confined geometries. Overall, tuning the AMPS:PEGDA composition allows us to span a wide range of water volume fractions and effective *C*_fix_ values. By comparing free‐standing and encapsulated conditions, we see that encapsulation provides a route to achieve higher *C*_fix_ than those attainable with the same composition in free‐standing form. The difference becomes more pronounced with increasing total polymer content and higher charged polymer fractions.

### 2.3 Polyelectrolyte Composition and Encapsulation Generate Distinct Nanostructure Characteristics

While *Φ*_w_ and *C*_fix_ offer insight into bulk properties, we next sought structural information to investigate possible differences arising from hydrogel formulation, both in free‐standing and encapsulated polyelectrolytes. Polyelectrolytes commonly exhibit phase‐separated nanostructures, with ionic domains embedded within the polymer network [34]. SAXS profiles were fitted using the Teubner–Strey (TS) model (Figures S18–S44), which is widely used for analyzing disordered, phase‐separated systems with short‐range spatial correlations. It is recognized to be particularly suitable for systems exhibiting broad scattering peaks due to disordered but periodically correlated domains, commonly found in swollen polymer networks and specifically in ion exchange membranes [24, 25, 26].

**FIGURE 4:**
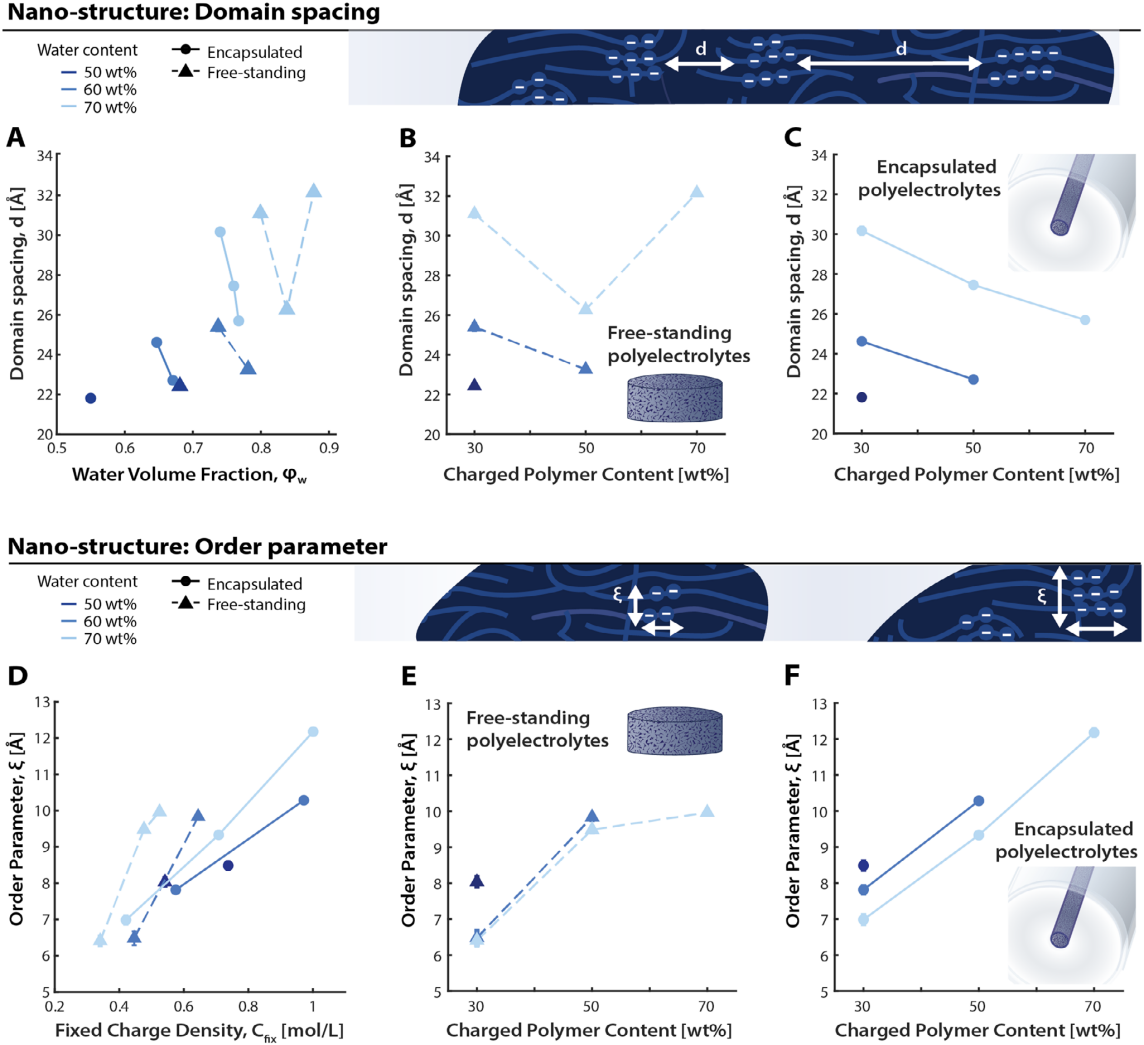
Nanoscale structural parameters obtained from Teubner–Strey analysis of SAXS data. Plot symbols (triangles and circles) and colors (blue hues) are consistent in all figure parts. (A) Domain spacing (d) vs. water volume fraction for free‐standing (triangles) and encapsulated (circles) polyelectrolytes. (B) d vs. charged polymer content for free‐standing polyelectrolytes. (C) d vs. charged polymer content for encapsulated polyelectrolytes. (D) Structural order parameter (ξ) as a function of *C*_fix_ for free‐standing (triangles) and encapsulated (circles) polyelectrolytes. (E) ξ vs. charged polymer content for free‐standing polyelectrolytes. (F) ξ vs. charged polymer content for encapsulated polyelectrolytes. Lines are added to guide the eye. Intensity profiles represent averages of *n* = 20 (encapsulated) and *n* = 8–10 (free‐standing) raw images.

Figure 4 summarizes the SAXS‐derived structural parameters. First, we investigated the domain spacing (*d*) as a function of *Φ*_w_. As seen in Figure 4A, there is a clear trend between the fraction of water and domain spacing, where the composition with the highest *Φ*_w_ resulted in the largest value of *d*. This is intuitive, as more swelling leads to a polymer network with ionic domains spaced further apart. Variation with charge density shows a different pattern. In free‐standing hydrogels, *d* showed a non‐monotonic trend with charge: as charged polymer content increased from 30 to 50 wt.%, *d* decreased, but at the highest charge (70 wt.% AMPS, and 70 wt.% water) *d* increased drastically (Figure 4B). We attribute this increase at high charge content to excessive swelling, i.e., beyond a certain charge density, the ionic domains grow farther apart due to the large water uptake. In contrast, in the encapsulated hydrogels (Figure 4C), *d* consistently decreased with increasing charged polymer content across the full parameter range.

In Figure 4D, where *ξ* is plotted against *C*_fix_, we note a clear trend: higher *C*_fix_ corresponds to enhanced structural organization and better‐connected ionic pathways. This pattern can be linked to increasing charged polymer content in both investigated cases; free‐standing (Figure 4E) and encapsulated polyelectrolyte hydrogels (Figure 4F). However, in free‐standing polyelectrolyte hydrogels of 30 wt.% polymer content, *ξ* seems to plateau at higher charge densities, similar to the plateau observed in *C*_fix_ for free‐standing hydrogels (Figure 3C). Again, this plateau is absent for encapsulated hydrogels across this examined parameter range, for both *ξ* (Figure 4F) and *C*_fix_ (Figure 3F). Across all conditions, *ξ* values remain smaller than *d*, indicating that the structural organization remains short‐range. In this framework, the ratio ξ/d is widely used as a measure of the degree of mesoscale order or “polydispersity”. Here, ξ/d falls into the range of 0.23–0.47 (see Figure S45), suggesting disordered systems with locally ordered but non‐crystalline domains, and consistent with previous reports of polyelectrolyte systems [35, 36].

Overall, SAXS analysis shows that free‐standing and encapsulated hydrogels follow different trends in structural parameters; while encapsulated polyelectrolytes show increasing order for increased charge content, we see a saturating effect for free‐standing polyelectrolytes at the highest charge fraction for both domain spacing and structural order. We attribute this saturation effect to excessive swelling at high charge content, which expands the average spacing between ionic domains and dilutes the charges. In contrast, in encapsulated hydrogels, we see no trace of saturation effects on domain spacing or structural order. This leads us to conclude that *C*_fix_ and *Φ*_w_ derived from measurements in free‐standing discs do not translate to the encapsulated case. Instead, these different trends support that confinement by encapsulation suppresses swelling and preserves a tighter nanostructure at high charge content. In addition, the clear link and strong positive Spearman correlation between both *d* and *Φ*_w_ (ρ = 0.714, p = 1.19E‐8), and between *ξ* and *C*_fix_ (ρ = 0.943, p = 1.37E‐23) for encapsulated polyelectrolytes support that numbers derived by polymer composition and geometrical constraints, provide reliable estimates of effective *Φ*_w_ and *C*_fix_.

### 2.4 Multiparameter Design Rules for Scalable Electrophoretic Delivery

**FIGURE 5:**
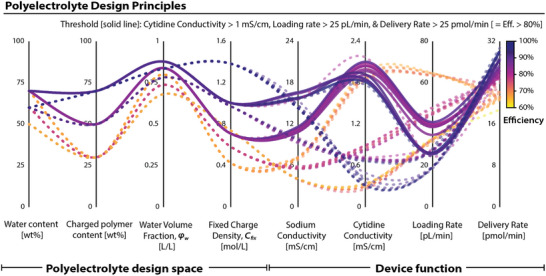
A multiparametric view of structure–property–function showing how material composition governs device‐level performance. The x‐axis categories represent distinct metrics, where lines connect values from the same formulation as a visual guide. Solid lines mark formulations exceeding threshold performance (cytidine conductivity > 1 mS cm^−^^1^, loading rate > 25 pL min^−^^1^, and delivery rate > 25 pmol min^−^^1^, corresponding to efficiency > 80%). The color gradient indicates delivery efficiency (dark blue highest). The map highlights a design window where sufficiently high *C*_fix_ and sufficient Φ_w_ coincide, balanced selectivity, resistance, and loading dynamics.

Overall, we found that although each performance metric is primarily driven by one aspect of composition, material properties, or nanostructure (Figure 2), many parameters are coupled. Figure 5 summarizes these multiparameter dependencies across the composition design space and how functional metrics co‐vary. In contrast to single parameter correlations, the map makes coupled trade‐offs explicit and highlights a narrow region where efficiency, loading, and resistance are simultaneously acceptable. Lines connect values from the same formulation as a visual guide, and solid lines mark formulations exceeding target thresholds. Since all delivery experiments were performed at the same applied current, the measured delivery rates and efficiency are effectively proportional across the dataset. Efficiency is encoded via the color scale, and the final axis presents delivery rate explicitly.

Importantly, high efficiency formulations appear on both sides of this conductivity separation, highlighting that efficiency and conductivity can be partially decoupled by balancing *C*_fix_ and *Φ_w_*. The key design trade‐off is therefore that higher *C*_fix_ improves selectivity and efficiency but must be balanced against increasing resistance and slower loading dynamics. The solid line subset identifies a composition window where sufficiently high *C*_fix_ and sufficient *Φ_w_* coincide, yielding high efficiency (> 80%), practical loading rates (> 25 pL min^−^^1^), and moderate resistance (cytidine conductivity > 1 mS cm^−^^1^). In this dataset, that balance occurs at charged polymer contents of 50–70 wt.% of total polymer combined with ∼70% water content.

The dose range investigated here is low, especially in the context of clinical systemic dosing, but can be biologically impactful for continuous local delivery. For reference, we previously demonstrated iontronic delivery of gemcitabine in a vascularized glioblastoma CAM model, which led to strong tumor growth inhibition over a five‐day treatment window at an iontronic current at 100 nA (delivery rates on the order of 10^1^ pmol min^−^^1^) [17]. The design framework presented here can further be used to preserve selectivity and scale dosing for specific targets. The delivery rates scale linearly with applied current and can be delivered via one larger or several parallel outlets, while manageable resistance as throughput is increased.

## 3 Conclusions

In this study, we performed structure–property–function mapping for drug‐scale ions in polyelectrolyte hydrogels to identify key design trade‐offs for drug delivery applications. We provide a method to derive *C*_fix_ under implant‐relevant conditions and show that encapsulation suppresses swelling and charge dilution, enabling higher effective *C*_fix_ than that in free‐standing films. Furthermore, we investigate multiparameter dependencies across the design space to derive polyelectrolyte design principles, especially tuned for drug delivery devices.

These results provide quantitative guidance for optimizing AMPS:PEGDA membranes for electrophoretic delivery, while clarifying the operational compromises between efficiency, resistance, and loading time. For the drug‐sized model ion across the investigated space, loading kinetics scale inversely with effective *C*_fix_, while transport efficiency increases with *C*_fix_ and nanostructural order, and conductivity is primarily governed by *Φ_w_* and domain spacing. Together, this highlights a permeability–selectivity balance that can be navigated by keeping *Φ_w_* high, and jointly tuning *C*_fix_.

The present analysis is based on a single model ion and idealized electrolyte conditions (matching physiological electrolyte concentrations); long‐term performance in vivo may be affected by material degradation or biofouling, which can alter both resistance and selectivity over time. Future work should therefore validate these trends for additional therapeutic ions and under in vivo‐relevant conditions. The design map presented here defines a practical starting point for that translation.

## 4 Experimental Section

### 4.1 Materials

2‐Acrylamido‐2‐methyl‐1‐propanesulfonic acid (AMPS) sodium salt solution (Mn 229.23 g mol^−1^, 50 wt.% in water), poly(ethylene glycol) diacrylate (PEGDA) (average Mn 575 g mol^−1^), the photoinitiator lithium phenyl‐2,4,6‐trimethylbenzoylphosphinate (LAP), 3‐(trimethoxysilyl)propyl methacrylate (A174 silane), and cytidine were purchased from Sigma–Aldrich (Sweden). UV transparent polyimide‐coated glass capillaries (TSH050375, Polymicro Technologies) with inner/outer 50 µm/375 µm diameters were purchased from CM Scientific.

### 4.2 Water Uptake of Free‐Standing Polyelectrolytes

Triplicates of approximately 200 µL of the pre‐polymerization mixtures were pipetted into 8 mm diameter PDMS wells attached to a plastic petri dish. The pre‐polymerization mixtures were exposed to UVA (350–410 nm, max. emission at 370 nm) for 30 min in a custom‐made photo exposure box. The polymerized membrane discs were placed in deionized water in 5 mL snap cap vials for three days, with water exchanged twice daily. Prior to measuring the wet polymer weight, the discs were gently dried with cleanroom paper to remove surface water. The wet mass was measured using an analytical microbalance (Sartorius BP 210 S). The dry mass was measured after drying under vacuum at 50°C for 3 days (Sanyo Gallenkamp Vacuum Oven).

### 4.3 Fabrication of Drug Delivery Devices

Devices were fabricated as previously reported [11]. In brief, UV transparent polyimide coated glass capillaries with inner/outer 50 µm/375 µm diameters were cut to a length of approximately 40 cm, and mounted to a Nordson EFD dispensing tip, and using a heat gun and tweezer to seal the connection. The inner glass surface was etched/activated by flushing 2 m KOH(aq) for 2 h, followed by flushing with deionized water for 10 min, and drying with nitrogen gas for 10 min. Next, capillaries were flushed with A174 silane (10% in toluene) for 1 h to promote adhesion of the polyelectrolyte. Ethanol was flushed for 20 min to remove excess silane, followed by drying with nitrogen gas for 20 min. Lastly, capillaries were filled with pre‐polymerization mixtures, sealed with Kapton tape at the capillary ends to prevent drying, and mounted onto a 3D printed holder, followed by exposure to UVA light for 3 h in a custom‐made photo exposure box. After polymerization, the capillaries were cut into approximately 20 mm pieces and soaked in 0.1 m NaCl(aq).

### 4.4 Device Characterization

The drug delivery devices (filled capillaries) were cut to a length of approximately 10 mm and mounted to 3D printed reservoirs. The reservoir with the micro‐catheter was placed into an Eppendorf tube filled with 150 µL of 1× PBS, which was used as the target electrolyte. 0.25 mm Ag/AgCl wires were used as source and counter electrodes, and an OctoStat30 8‐channel potentiostat (Ivium Technologies) was used to apply current, and measure potential, throughout all device characterization steps. First, to determine sodium conductivity, the source reservoir was filled with fresh 0.1 m NaCl(aq), and a constant current of 50 nA was applied for 2 h, followed by a series of current levels of 25, 50, 75, and 100 nA (3 min each) (Figures S10–S14).

Second, for collecting cytidine transport data, the target electrolyte was changed to 0.1 m cytidine (adjusted to pH 4.20), and a constant current of 50 nA was applied again. After an initial increase of device resistance during drug loading, a plateau (where voltage remained nearly constant) was reached. Thus, loading time for each device was determined from start to when the maximum resistance was reached (5‐17 h) shortly after the linear rise (Figures S3–S9). The drug loading rate (pL min^−1^) was calculated by converting the reciprocal of the loading time per unit length (hours mm^−1^) into a volumetric flux, accounting for capillary cross‐sectional area, and expressing the result in pL min^−1^.

And finally, for characterization of cytidine delivery rates, target and source electrolytes were again exchanged to fresh solutions. A constant current of 50 nA was applied for 24 h (Figures S3–S9), and the target electrolytes were saved for spectrophotometric analysis. Absorbance of the target electrolyte containing 1× PBS and cytidine was measured using an Implen NP80 microvolume nanophotometer at 271 nm. A calibration curve (Figure S46) was used to determine concentration, further translated to delivery rates (pmol min^−1^) and efficiency. Equations in SI.

Sodium transport was measured across the full dataset of 11 polyelectrolyte compositions. For cytidine transport, the dataset is limited to compositions with manageable device resistance. Devices that did not reach the target current of 50 nA at the potentiostat limit of 10 V (low *Φ*_w_ combined with high *C*_fix_) were excluded from further analysis. Consequently, cytidine performance metrics are reported for a subset of seven compositions within a practically accessible resistance range. For each composition, 6–8 replicates were used (outliers were excluded).

### 4.5 SAXS Characterization

Thin‐walled quartz glass capillaries (outer diameter 1.5 mm, 10 µm wall thickness, Charles Supper Company, UK) were first treated with UV ozone, followed by silanization (A174 silane) overnight by vapor deposition at room temperature. The center of the capillaries was filled with pre‐polymerization mixture, exposed for 20 min in UVA. After polymerization, the glass capillaries were soaked in 0.1 m NaCl(aq) for a total of 7 days prior to measurement. Unfortunately, the strong swelling of two polyelectrolyte compositions at the highest *C*_fix_, fractured the glass during hydration, resulting in a dataset of five compositions for the analysis of encapsulated samples.

Free‐standing samples were prepared according to the protocol developed for water uptake characterization, and stored in 0.1 m NaCl(aq) for 6 days. Measurements were performed at the ForMAX beamline at MAX IV Laboratory (Lund, Sweden). The beamline was operated at an energy of 16.15 keV, and scattering patterns were recorded using an Eiger2 × 4 M detector (Detectric Ltd) positioned at a sample‐to‐detector distance of 1.255 m, covering an angular range of 0.01 ≤ q ≤ 0.77 Å^−^^1^. Samples were mounted on custom‐designed 3D‐printed holders, and data were collected by scanning the sample along the sample while recording a single exposure at each position. This approach ensured that fresh material was continuously exposed to the X‐ray beam, thereby minimizing radiation damage and improving data representativeness. Raw 2D scattering images were azimuthally averaged and reduced to 1D intensity profiles using a custom Python script. The averaged intensity profiles were calculated from 20 raw images for the encapsulated samples and 8–10 raw images for the free‐standing samples. Scattering from background samples was subtracted from that of the polyelectrolyte samples to obtain I(q) profiles. Curve fitting was performed in SasView (version 6.0) using the Teubner–Strey model to extract the domain spacing (*d*) and structural correlation length (*ξ*). All corresponding equations, raw data, curve fits, and residuals are provided in Figures S18–S44.

### 4.6 Statistical Analysis

A Spearman rank correlation matrix was calculated in GraphPad Prism 10.0.2 to test for significant monotonic pair‐wise relationships between all variables (Figure S2). Two‐tailed *p*‐values were calculated and considered significant at *p* < 0.05. Simple linear regression was performed in MATLAB R2024a.

## Conflicts of Interest

M.B., D.T.S., I. B.W. and T.A.S. are shareholders in the small, researcher controlled intellectual property company OBOE IPR AB (oboeipr.com). OBOE IPR AB owns patents related to this research, and is a holding company for subsidiary Iontronics AB.

## Supporting information




**Supporting File**: advs74843‐sup‐0001‐SuppMat.pdf

## Data Availability

The data that support the findings of this study are available from the corresponding author upon reasonable request.

## References

[1] E. Magill , S. Demartis , E. Gavini , et al., “Solid Implantable Devices for Sustained Drug Delivery,” Advanced Drug Delivery Reviews 199 (2023): 114950, 10.1016/j.addr.2023.114950. 37295560

[2] D. T. Manallack , “The pKa Distribution of Drugs: Application to Drug Discovery,” Perspectives in Medicinal Chemistry 1 (2007): 25–38. 19812734 PMC2754920

[3] R. Pirie , H. A. Stanway‐Gordon , H. L. Stewart , et al., “An Analysis of the Physicochemical Properties of Oral Drugs from 2000 to 2022,” RSC Medicinal Chemistry 15 (2024): 3125–3132, 10.1039/D4MD00160E. 39309358 PMC11411612

[4] J. Li and D. J. Mooney , “Designing Hydrogels for Controlled Drug Delivery,” Nature Reviews Materials 1 (2016): 16071, 10.1038/natrevmats.2016.71. PMC5898614 29657852

[5] T. Arbring Sjöström , M. Berggren , E. O. Gabrielsson , et al., “A Decade of Iontronic Delivery Devices,” Advanced Materials Technologies 3 (2018): 1700360, 10.1002/admt.201700360.

[6] J. Isaksson , P. Kjäll , D. Nilsson , N. Robinson , M. Berggren , and A. Richter‐Dahlfors , “Electronic Control of Ca^2+^ Signalling in Neuronal Cells Using an Organic Electronic Ion Pump,” Nature Materials 6 (2007): 673–679, 10.1038/nmat1963. 17643105

[7] K. Tybrandt , K. C. Larsson , S. Kurup , et al., “Translating Electronic Currents to Precise Acetylcholine–Induced Neuronal Signaling Using an Organic Electrophoretic Delivery Device,” Advanced Materials 21 (2009): 4442–4446, 10.1002/adma.200900187.

[8] M. Jia , L. Luo , and M. Rolandi , “Correlating Ionic Conductivity and Microstructure in Polyelectrolyte Hydrogels for Bioelectronic Devices,” Macromolecular Rapid Communications 43 (2022): 2100687, 10.1002/marc.202100687. 35020249

[9] M. Seitanidou , J. F. Franco‐Gonzalez , T. A. Sjöström , I. Zozoulenko , M. Berggren , and D. T. Simon , “pH Dependence of γ‐Aminobutyric Acid Iontronic Transport,” The Journal of Physical Chemistry B 121 (2017): 7284–7289, 10.1021/acs.jpcb.7b05218. 28741949

[10] C. M. Proctor , A. Slézia , A. Kaszas , et al., “Electrophoretic Drug Delivery for Seizure Control,” Science Advances 4 (2018): aau1291, 10.1126/sciadv.aau1291. PMC6114990 30167463

[11] I. Bernacka‐Wojcik , M. Huerta , K. Tybrandt , et al., “Implantable Organic Electronic Ion Pump Enables ABA Hormone Delivery for Control of Stomata in an Intact Tobacco Plant,” Small 15 (2019): 1902189, 10.1002/smll.201902189. 31513355

[12] A. Roy , A. Bersellini Farinotti , T. Arbring Sjöström , et al., “Electrophoretic Delivery of Clinically Approved Anesthetic Drug for Chronic Pain Therapy,” Advanced Therapeutics 6 (2023): 2300083, 10.1002/adtp.202300083.

[13] L. Waldherr , M. Seitanidou , M. Jakešová , et al., “Targeted Chemotherapy of Glioblastoma Spheroids With an Iontronic Pump,” Advanced Materials Technologies 6 (2021): 2001302, 10.1002/admt.202001302. 34195355 PMC8218220

[14] H. Yoo , S.‐B. Kang , J. Kim , et al., “Ionic Diode‐Based Drug Delivery System,” Advanced Materials 37 (2025): 2412377, 10.1002/adma.202412377. 39718239

[15] H. Li , N. Asefifeyzabadi , K. Schorger , et al., “Remote‐Controlled Wireless Bioelectronics for Fluoxetine Therapy to Promote Wound Healing in a Porcine Model,” Advanced Materials Technologies 10 (2025): 70039, 10.1002/admt.202500040.

[16] D. J. Poxson , E. O. Gabrielsson , A. Bonisoli , et al., “Capillary‐Fiber Based Electrophoretic Delivery Device,” ACS Applied Materials & Interfaces 11 (2019): 14200–14207, 10.1021/acsami.8b22680. 30916937

[17] V. Handl , L. Waldherr , T. Arbring Sjöström , et al., “Continuous Iontronic Chemotherapy Reduces Brain Tumor Growth in Embryonic Avian In Vivo Models,” Journal of Controlled Release 369 (2024): 668–683, 10.1016/j.jconrel.2024.03.044. 38548064

[18] G. M. Geise , D. R. Paul , and B. D. Freeman , “Fundamental Water and Salt Transport Properties of Polymeric Materials,” Progress in Polymer Science 39 (2014): 1–42, 10.1016/j.progpolymsci.2013.07.001.

[19] T. Luo , S. Abdu , and M. Wessling , “Selectivity of Ion Exchange Membranes: A Review,” Journal of Membrane Science 555 (2018): 429–454, 10.1016/j.memsci.2018.03.051.

[20] D. Kitto and J. Kamcev , “The Need for Ion‐Exchange Membranes With High Charge Densities,” Journal of Membrane Science 677 (2023): 121608, 10.1016/j.memsci.2023.121608.

[21] J. C. Díaz and J. Kamcev , “Ionic Conductivity of Ion‐Exchange Membranes: Measurement Techniques and Salt Concentration Dependence,” Journal of Membrane Science 618 (2021): 118718, 10.1016/j.memsci.2020.118718.

[22] S. Prevost , T. Lopian , M. Pleines , O. Diat , and T. Zemb , “Small‐Angle Scattering and Morphologies of Ultra‐Flexible Microemulsions,” Journal of Applied Crystallography 49 (2016): 2063–2072, 10.1107/S1600576716016150. 27980512 PMC5139994

[23] S. S. Welborn and E. Detsi , “Small‐Angle X‐ray Scattering of Nanoporous Materials,” Nanoscale Horizons 5 (2019): 12–24, 10.1039/C9NH00347A.

[24] M. Teubner and R. Strey , “Origin of the Scattering Peak in Microemulsions,” The Journal of Chemical Physics 87 (1987): 3195–3200, 10.1063/1.453006.

[25] R. J. Hickey , T. M. Gillard , M. T. Irwin , T. P. Lodge , and F. S. Bates , “Structure, Viscoelasticity, and Interfacial Dynamics of a Model Polymeric Bicontinuous Microemulsion,” Soft Matter 12 (2015): 53–66, 10.1039/C5SM02009C. 26439750

[26] Y. Zhao , K. Yoshimura , T. Motegi , A. Hiroki , A. Radulescu , and Y. Maekawa , “Three‐Component Domains in the Fully Hydrated Nafion Membrane Characterized by Partial Scattering Function Analysis,” Macromolecules 54 (2021): 4128–4135, 10.1021/acs.macromol.1c00587.

[27] J. Morris , D. G. Wishka , O. D. Lopez , et al., “F‐aza‐T‐dCyd (NSC801845), a Novel Cytidine Analog, in Comparative Cell Culture and Xenograft Studies With the Clinical Candidates T‐dCyd, F‐T‐dCyd, and Aza‐T‐dCyd,” Molecular Cancer Therapeutics 20 (2021): 625–631, 10.1158/1535-7163.MCT-20-0738. 33811149 PMC8030693

[28] M. Seitanidou , K. Tybrandt , M. Berggren , and D. T. Simon , “Overcoming Transport Limitations in Miniaturized Electrophoretic Delivery Devices,” Lab on a Chip 19 (2019): 1427–1435, 10.1039/C9LC00038K. 30875418

[29] T. Arbring Sjöström , A. Jonsson , E. O. Gabrielsson , M. Berggren , D. T. Simon , and K. Tybrandt , “Miniaturized Ionic Polarization Diodes for Neurotransmitter Release at Synaptic Speeds,” Advanced Materials Technologies 5 (2020), 10.1002/admt.201900750.

[30] J. Kamcev , D. R. Paul , and B. D. Freeman , “Effect of Fixed Charge Group Concentration on Equilibrium Ion Sorption in Ion Exchange Membranes,” Journal of Materials Chemistry A 5 (2017): 4638–4650, 10.1039/C6TA07954G.

[31] N. Yan , D. R. Paul , and B. D. Freeman , “Water and Ion Sorption in a Series of Cross‐Linked AMPS/PEGDA Hydrogel Membranes,” Polymer 146 (2018): 196–208, 10.1016/j.polymer.2018.05.021.

[32] N. Yan , R. Sujanani , J. Kamcev , et al., “Influence of Fixed Charge Concentration and Water Uptake on Ion Sorption in AMPS/PEGDA Membranes,” Journal of Membrane Science 644 (2022): 120171, 10.1016/j.memsci.2021.120171.

[33] T. Cano , H. Na , J.‐Y. Sun , and H.‐Y. Kim , “Swelling Kinetics of Constrained Hydrogel Spheres,” Soft Matter 19 (2023): 8820–8831, 10.1039/D3SM01228J. 37947035

[34] L. Boldon , F. Laliberte , and L. Liu , “Review of the Fundamental Theories behind Small Angle X‐ray Scattering, Molecular Dynamics Simulations, and Relevant Integrated Application,” Nano Reviews 6 (2015): 25661, 10.3402/nano.v6.25661. 25721341 PMC4342503

[35] S. S. He and C. W. Frank , “Facilitating Hydroxide Transport in Anion Exchange Membranes via Hydrophilic Grafts,” Journal of Materials Chemistry A 2 (2014): 16489–16497, 10.1039/C4TA02942A.

[36] M. L. Le , D. J. Grzetic , K. T. Delaney , et al., “Electrostatic Interactions Control the Nanostructure of Conjugated Polyelectrolyte–Polymeric Ionic Liquid Blends,” Macromolecules 55 (2022): 8321–8331, 10.1021/acs.macromol.2c01142.

