# Supplementary material for: Polyelectrolyte Design Principles for Electrophoretic Drug Delivery
Source: Adv Sci (Weinh). 2026 Mar 15;13(30):e22981. doi: 10.1002/advs.202522981 (PMC13248822; doi:10.1002/advs.202522981)
Supplement: Supplementary file 1 — Supporting File: advs74843‐sup‐0001‐SuppMat.pdf [file ADVS-13-e22981-s001.pdf]

SUPPORTING INFORMATION

Polyelectrolyte Design Principles for Electrophoretic Drug Delivery

*Helena Saarela Unemo[1], Iwona Bernacka-Wojcik[1], Lingkai Zhu[1], Marle E.J. Vleugels[1], Moa E. Hörberg[1], Caroline Lindholm[1], Magnus Berggren[1,2], Daniel T. Simon[1], Theresia Arbring Sjöström[1,3]***

[1] Laboratory of Organic Electronics, Dept. of Science and Technology, Linköping University, 601 74 Norrköping, Sweden

[2] Wallenberg Initiative Materials Science for Sustainability, Department of Science and Technology, Linköping University, 60 174 Norrköping, Sweden

[3] Department of Biomedical Engineering, Linköping University, 581 85 Linköping, Sweden

** Corresponding author: theresia.arbring.sjostrom@liu.se

Table of Contents

1. Polyelectrolyte Composition Matrix 2
2. Experimental Setup 2
3. Parametric Spearman Correlation Analysis 3
4. Device Characterization: Cytidine 3
5. Device Characterization: Sodium 7
6. Water Volume Fraction, and Fixed Charge Density in Free-standing Polyelectrolytes 9
7. Water Volume Fraction, and Fixed Charge Density in Encapsulated Polyelectrolytes 10
8. SAXS 11
8.1. Theory 11
8.2. 1D Raw Data and SasView fits 11
9. Delivery Rate Determination 19
References 20

1. Polyelectrolyte Composition Matrix

Table S1. AMPS:PEGDA material compositions.

Water content (wt%)	Charged polymer content, AMPS (wt%)	Neutral polymer content, PEGDA (wt%)	IEC (mmol/g)
75	30	70	1.31
70	30	70	1.31
60	30	70	1.31
50	30	70	1.31
75	40	60	1.74
70	40	60	1.74
60	40	60	1.74
50	40	60	1.74
75	50	50	2.18
70	50	50	2.18
60	50	50	2.18
50	50	50	2.18
75	60	40	2.62
70	60	40	2.62
60	60	40	2.62
50	60	40	2.62
75	70	30	3.05
70	70	30	3.05
60	70	30	3.05
50	70	30	3.05

2. Experimental Setup

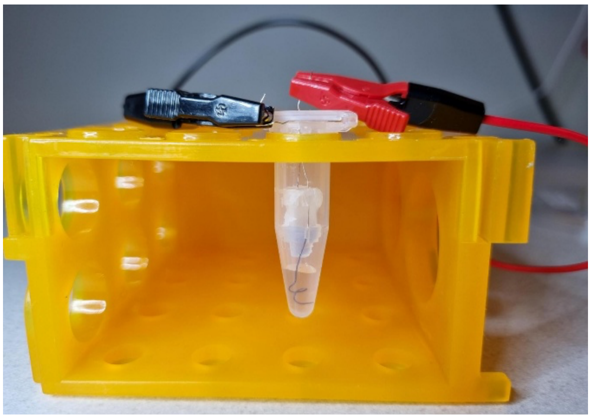

Figure S1. *Experimental two-electrode (0.25 mm Ag/AgCl wires) set-up for ion conductivity and delivery experiments.*

3. Parametric Spearman Correlation Analysis

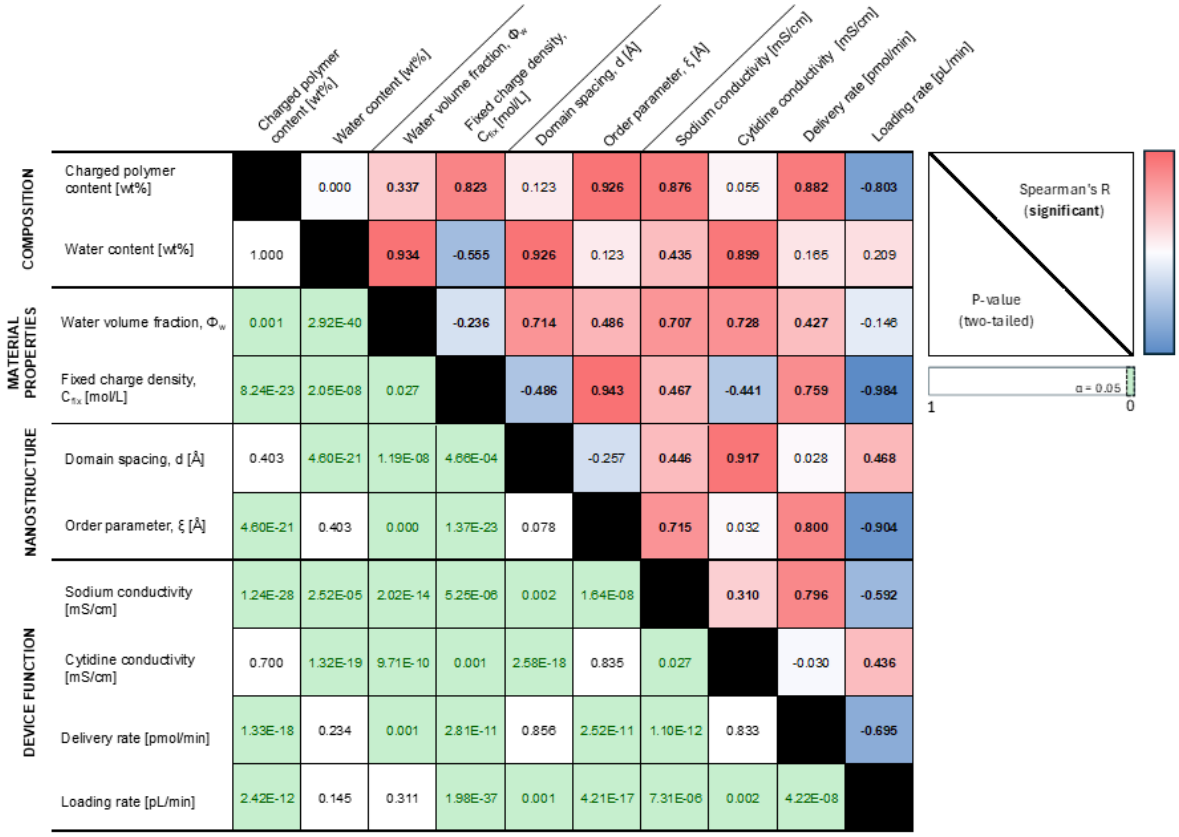

Figure S2. *Spearman's correlations heat maps illustrating the pairwise relationships between measured parameters. Correlation coefficients are shown for statistically significant relationships ($p < 0.05$, two-tailed test).*

4. Device Characterization: Cytidine

Cytidine loading rate was determined from the loading curves (dotted lines indicate the time of loading for each device). Cytidine conductivity was calculated from the delivery curves.

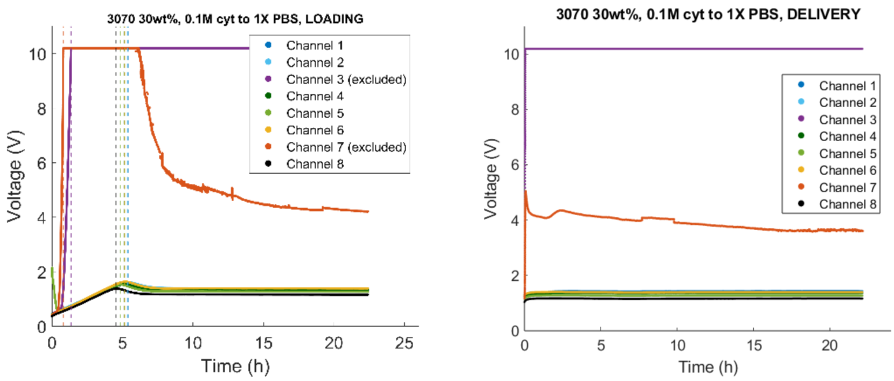

Figure S3. *Cytidine loading curve with indicated loading time (left) and delivery curve (right) for devices with AMPS:PEGDA 30:70, 30 wt% total polymer.*

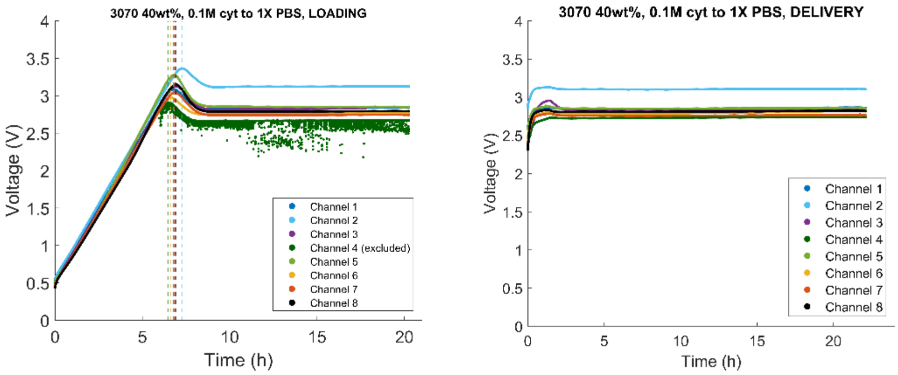

Figure S4. *Cytidine loading curve with indicated loading time (left) and delivery curve (right) for devices with AMPS:PEGDA 30:70, 40 wt% total polymer.*

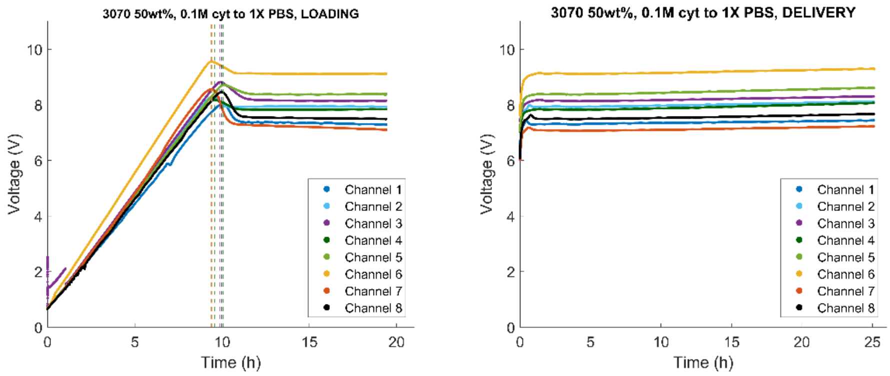

Figure S5. *Cytidine loading curve with indicated loading time (left) and delivery curve (right) for devices with AMPS:PEGDA 30:70, 50 wt% total polymer.*

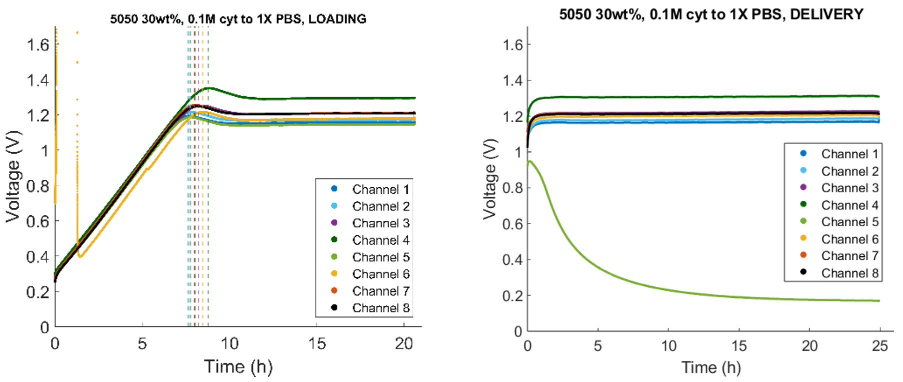

Figure S6. *Cytidine loading curve with indicated loading time (left) and delivery curve (right) for devices with AMPS:PEGDA 50:50, 30 wt% total polymer.*

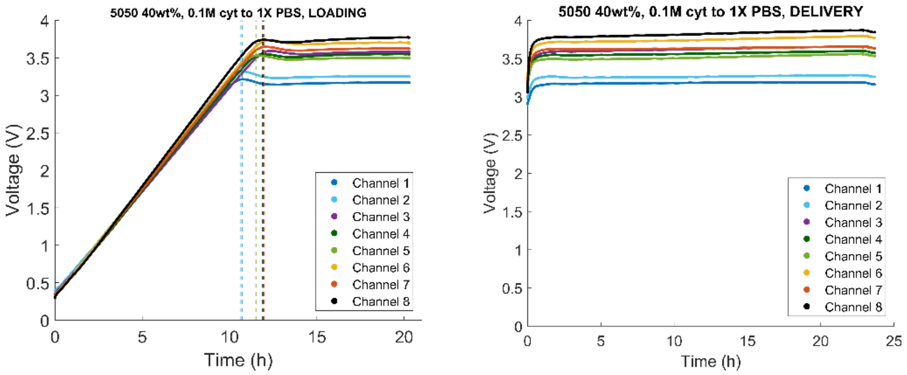

Figure S7. *Cytidine loading curve with indicated loading time (left) and delivery curve (right) for devices with AMPS:PEGDA 50:50, 40 wt% total polymer.*

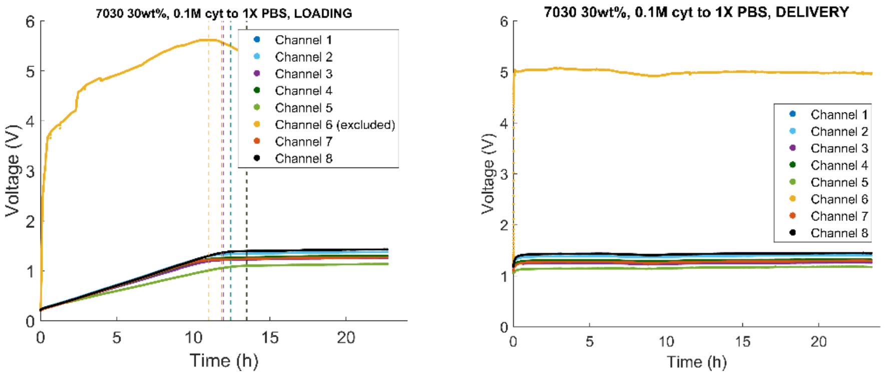

Figure S8. *Cytidine loading curve with indicated loading time (left) and delivery curve (right) for devices with AMPS:PEGDA 70:30, 30 wt% total polymer.*

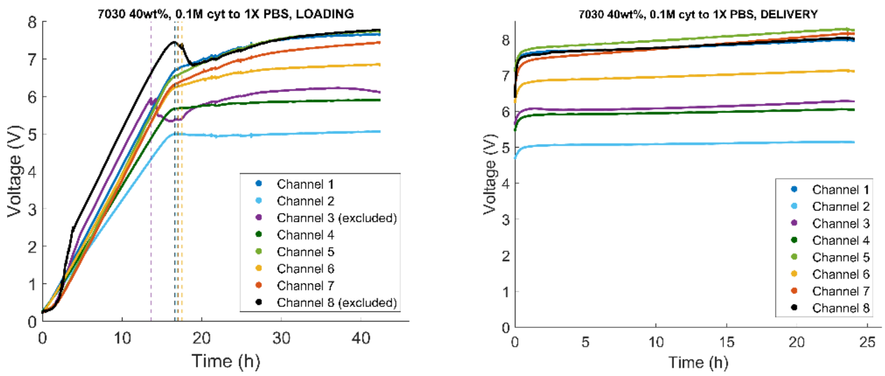

Figure S9. *Cytidine loading curve with indicated loading time (left) and delivery curve (right) for devices with AMPS:PEGDA 70:30, 40 wt% total polymer.*

5. Device Characterization: Sodium

Voltage was recorded throughout. From 0 to 2 h a constant current of 50 nA was applied. Thereafter, stepwise current levels of 25, 50, 75, and 100 nA were applied in sequence, each for 3 min.

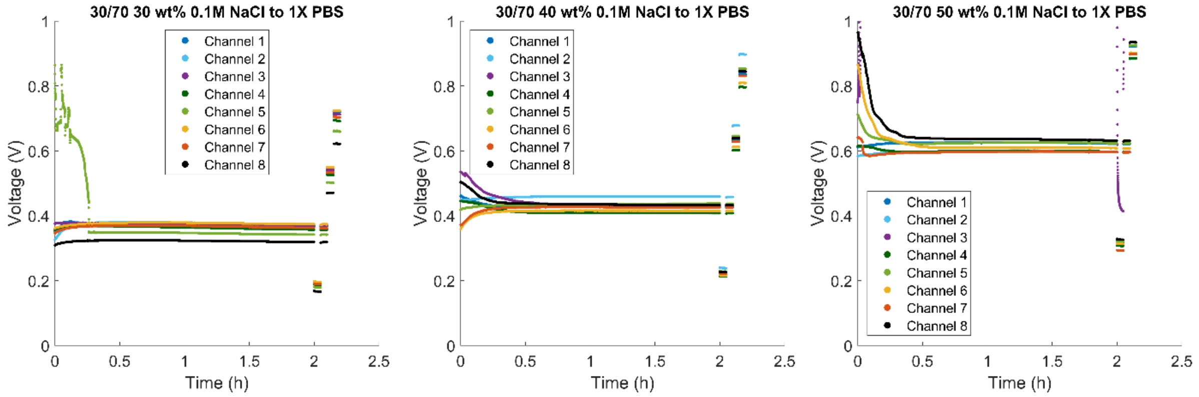

Figure S10. *Voltage vs time plots for AMPS:PEGDA 30:70 polyelectrolytes (30 wt%, 40 wt%, and 50 wt% total polymer).*

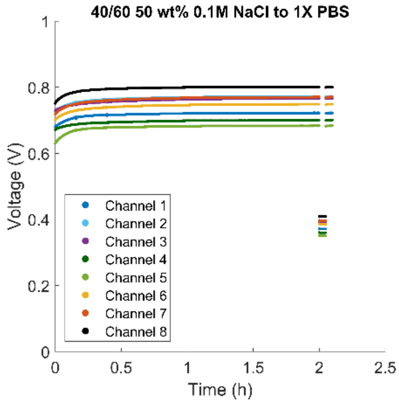

Figure S11. *Voltage vs time plots for AMPS:PEGDA 40:60, 50 wt% total polymer.*

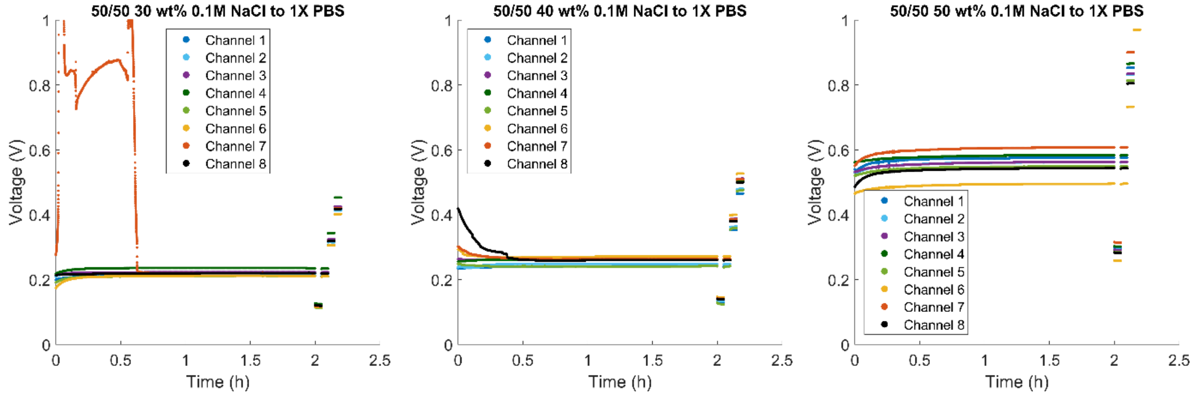

Figure S12. *Voltage vs time plots for AMPS:PEGDA 50:50 polyelectrolytes (30 wt%, 40 wt%, and 50 wt% total polymer).*

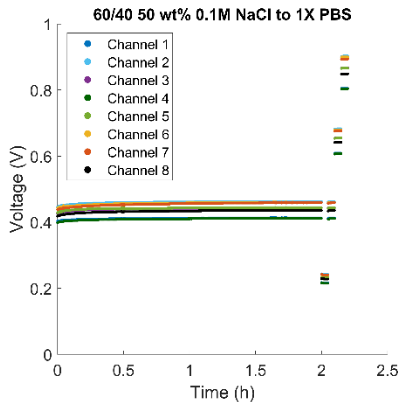

Figure S13. *Voltage vs time plots for AMPS:PEGDA 60:40, 50 wt% total polymer.*

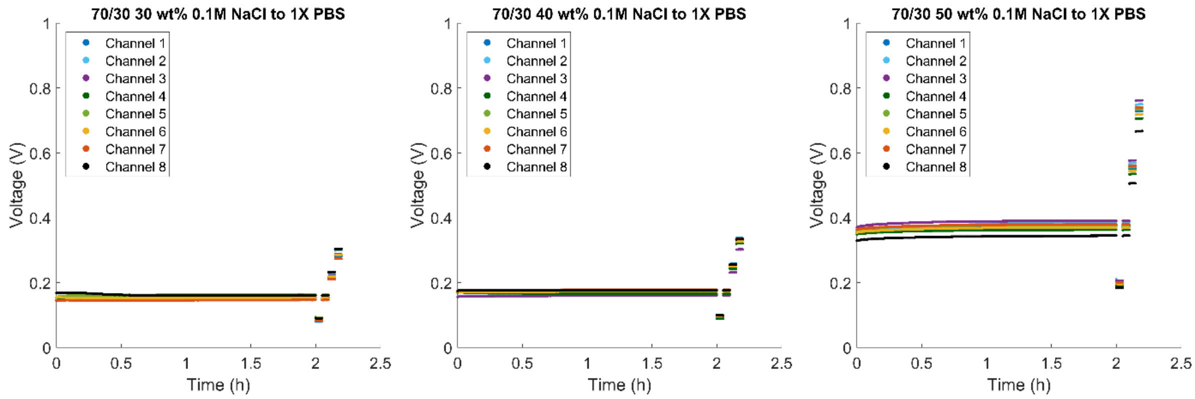

Figure S14. *Voltage vs time plots for AMPS:PEGDA 70:30 polyelectrolytes (30 wt%, 40 wt%, and 50 wt% total polymer).*

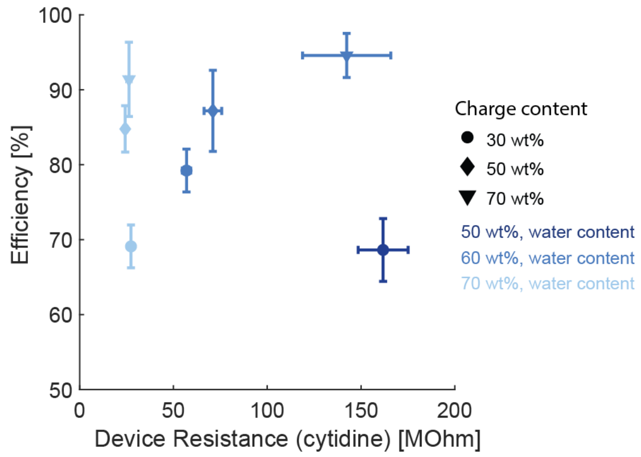

Figure S15. *Delivery efficiency vs device resistance during cytidine delivery.*

6. Water Volume Fraction, and Fixed Charge Density in Free-standing Polyelectrolytes

An experimental measure of the capacity of the dry polymer to absorb water, is the water uptake (w_u), defined as

$$w_u = \frac{m_w}{m_{dry}} = \frac{m_{wet} - m_{dry}}{m_{dry}}$$

where m_w is the mass of water, m_{wet} is the mass of the hydrated polymer, m_{dry} the mass of dry polymer. Water uptake measurements are generally performed in freestanding films[1,2] or discs[3] soaked in deionized water, where the osmotic pressure is effectively zero, allows the polyelectrolyte hydrogel to swell to their maximum capacity. Furthermore, by considering the density ratio between water and polymer, the water volume fraction (Φ_w), defined as the fraction of water volume, V_w, to total volume of hydrated polymer, V_{tot}

$$\Phi_w = \frac{V_w}{V_{tot}} = \frac{w_u}{w_u + (\rho_w/\rho_p)}$$

For freestanding polyelectrolytes, V_{tot} is a sum of the solvent added before polymerization and additional solvent uptaken after polymerization. For enclosed systems

$$V_{tot,enclosed} = V_{polymer} + V_{solvent,enclosed} = \frac{m_{polymer}}{\rho_p} + \frac{m_{solvent}}{\rho_s}$$

where ρ_s is the density of solvent, $m_{solvent}$ mass of solvent added to the enclosed volume before polymerization, and where ρ_p is the polymer density, $m_{polymer}$ is the polymer mass added to the enclosed volume before polymerization. The resulting water volume fractions were derived by using polymer densities extrapolated by Yan et al.[2] (Fig. S16) For these compositions, the density range falls within 1.3-1.4 kg L^{-1}.

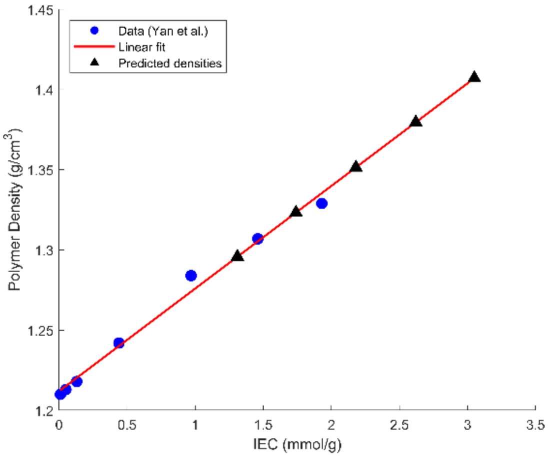

***Figure S16**. Linear fit on polymer densities from Yan et al.[2] extrapolated to IEC values used in this study.*

Beyond hydration and its influence on mesh size, the incorporation of charge units into the polymer network introduces additional property that influences polymer structure and function. A measure of the total content of charged groups in the polyelectrolyte is ion exchange capacity (IEC). IEC is a theoretical measure from the membrane composition and is defined as the number of charges units per gram of dry polymer (mmol g^{-1}):

$$IEC = \frac{n_{fix}}{m_{dry}} = \frac{AMPS\,[mmol]}{AMPS\,[g] + PEGDA\,[g]}$$

Crucially, IEC represents the inherent charge capacity of the material prior to swelling, serving as a fundamental property of the dehydrated blend. Since the charge units are highly hydrophilic, polyelectrolytes tend to swell excessively when submerged in water-based electrolytes, if physical space and crosslinking allow.

The resulting fixed charge density (C_{fix}) defines the density of immobile charged groups in the hydrated polyelectrolyte network (mol L^{-1}). C_{fix} and is derived from w_u, IEC, and Φ_w.

$$C^{fix}_{\text{free}} = \frac{n_{\text{fix}}}{V_{\text{tot}}} = \frac{IEC \cdot \rho_w}{w_u} \Phi_w = \frac{IEC \cdot \rho_w}{w_u + (\rho_w \cdot \rho_p{}^{-1})}$$

Therefore, while IEC quantifies the total charge in the dry material, C_{fix} provides the effective concentration of these charges within the swollen hydrogel, directly impacting its functional performance in drug transport.

7. Water Volume Fraction, and Fixed Charge Density in Encapsulated Polyelectrolytes

To better approximate the C_{fix} when swelling is restricted within a closed volume, we reformulated the water uptake as a water-to-polymer mass ratio ($m_w \cdot m_p{}^{-1}$). Water volume fraction for encapsulated devices, were furthermore theoretically derived, by using the same polymer densities as previously, leading to an estimate of the effective C_{fix} under closed conditions, of

$$C^{fix}_{\text{closed}} = \frac{n_{fix}}{V_{tot}} = \frac{IEC \cdot \rho_w}{m_w \cdot m_p{}^{-1}} \Phi_{w,closed} = \frac{IEC \cdot \rho_w}{(m_w \cdot m_p{}^{-1}) + (\rho_w \cdot \rho_p{}^{-1})}$$

For C^{fix}_{closed}, it is assumed that the total volume is filled and remained during polymerization and assumes ideal polymerization (i.e. no loss of uncrosslinked monomers during soaking). It should be noted that the no-loss assumption might underestimate the water volume fraction and may therefore overestimate the true effective C_{fix}.

The C^{fix}_{closed} assumption has limitations, particularly at extreme polymer concentrations and charge contents. At very high polymer weight fractions (corresponding to extremely dense polyelectrolyte hydrogel) the fixed charge concentration reached asymptotically high values, which are not physically plausible. This behaviour is illustrated in Figure S17 A, where the fixed charge concentration increases exponentially for high polymer wt%. Similarly, at very high charge content with minimal or no crosslinking, i.e., low PEGDA fraction, the system would consist of short polymer chains lacking the structural integrity of a true membrane. Without sufficient crosslinking, the membrane cannot maintain its form, rendering the C^{fix}_{closed} prediction invalid in such cases. As shown in Figure S17 C, predicted C^{fix}_{closed} continues to increase linearly with charge content, even beyond realistic structural limits. Note that the tested compositions (in Table S1) do not approach extreme limits where the C^{fix}_{closed} prediction becomes invalid.

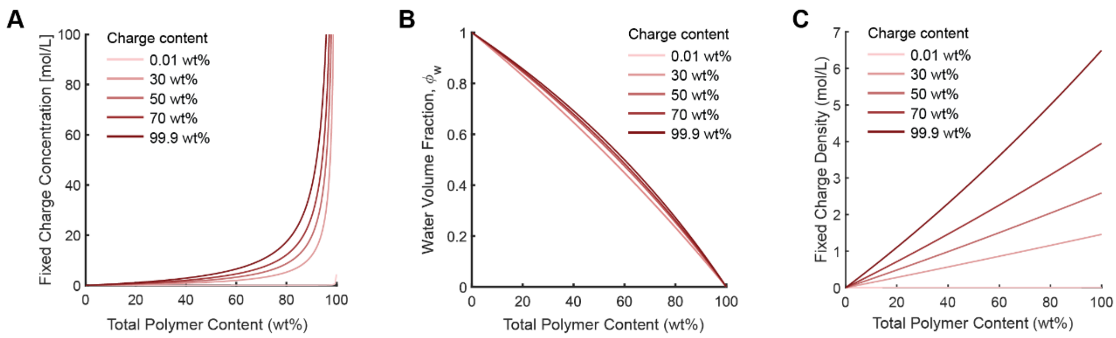

Figure S17. *Fixed charge concentration (A), water volume fraction (B), and fixed charge density for encapsulated systems (C). Charge content range: 0.01 – 99.9 wt%, total polymer content range: 0 – 100 wt%.*

8. SAXS

8.1. Theory

Small-angle X-ray scattering (SAXS) was conducted on selected material compositions to determine the order parameter (ξ) and the characteristic domain spacing (d). To extract ξ and d, the scattering profiles were fitted using the Teubner–Strey (TS) model:

$$I(q) = \frac{1}{a_2 + c_1 q^2 + c_2 q^4}$$

where $I(q)$ is the background corrected scattering intensity, q is the scattering vector ($q = 4\pi \sin(\vartheta)/\lambda$ for scattering angle θ and incident X-ray wavelength λ), and a_1, c_1, and c_2 are fitting coefficients. ξ and d are derived from the fitting coefficients of the TS model:

$$\xi = \left[\frac{1}{2}\left(\frac{a_2}{c_2}\right)^{1/2} + \frac{1}{4}\left(\frac{c_1}{c_2}\right)\right]^{-1/2}$$

and

$$d = 2\pi\left[\frac{1}{2}\left(\frac{a_2}{c_2}\right)^{1/2} - \frac{1}{4}\left(\frac{c_1}{c_2}\right)\right]^{-1/2}$$

Here, the order parameter (ξ) reflects the extent of structural organization and connectivity between hydrated domains, while the domain spacing (d) corresponds to the average distance between ionic clusters.[4,5]

8.2. 1D Raw Data and SasView fits

1D plots for samples below were averaged and the averaged background was subtracted before fitting the data in SasView. A glass capillary filled with 0.1 M NaCl(aq) (Figure S18) was used as the background for capillary samples. For free-standing samples, the background corresponded to 0.1 M NaCl(aq), obtained by subtracting the scattering from an air-filled capillary (Figure S19) from that of a capillary filled with 0.1 M NaCl(aq), thereby isolating the solvent contribution. For model fitting in SasView only the key parameters relevant to the primary structural features were included in the fitting: the domain spacing d, correlation length ξ, scale factor, and background. Initial parameter guesses were chosen: the domain spacing (d) was estimated using $d = 2\pi/q^*$, where $q^*$is the position of the primary scattering peak, and the structural order parameter (ξ) was initially set to 10 Å. The fitting procedure prioritized achieving randomly distributed residuals with magnitudes below 1 (see residual plots below). Consequently, the q-range for fitting was narrowed (0.15–0.36 $Å^{-1}$), tailored for each sample to focus on the primary peak region and maintain reliable residual behavior.

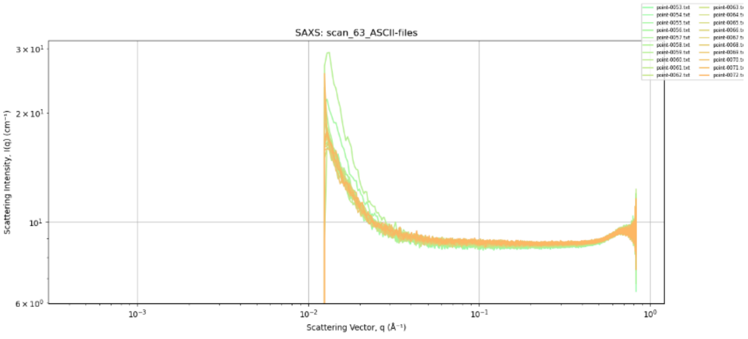

Figure S18. *1D plot for a glass capillary filled with 0.1M NaCl(aq).*

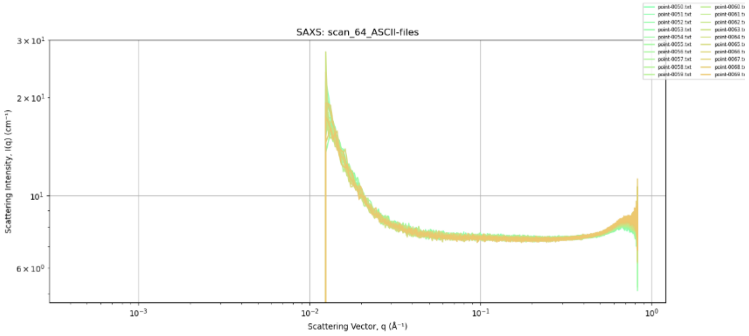

Figure S19. *1D plot for an empty (air-filled) glass capillary.*

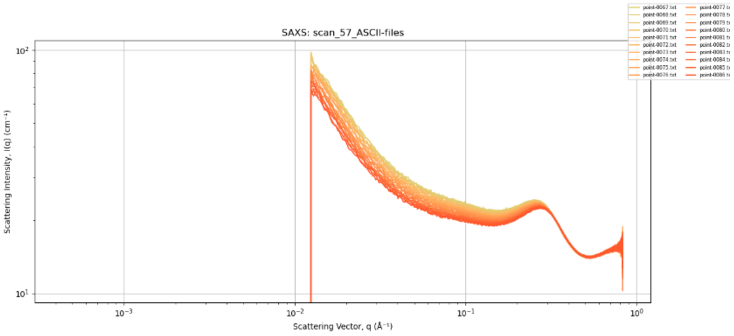

Figure S20. *Raw 1D plot for AMPS:PEGDA 30:70, 50 wt% total polymer in a glass capillary.*

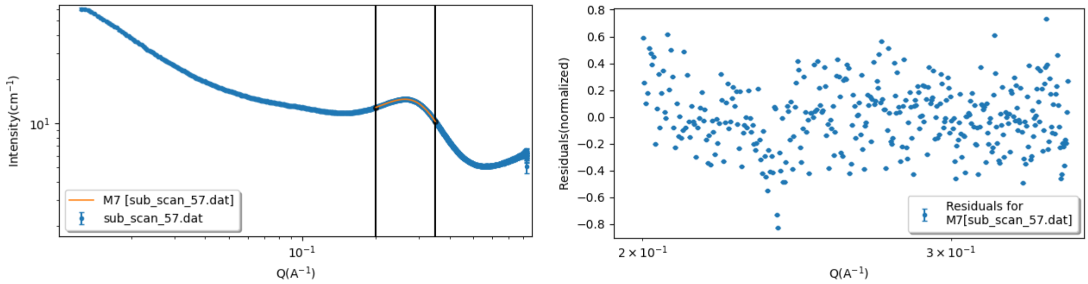

Figure S21. *Fitting of the background-subtracted 1D plot using the Teubner-Strey model in SasView 6.0 with a Q-range of 0.20-0.35 Å (left) and the corresponding residuals (right). Sample: AMPS:PEGDA 30:70, 50 wt% total polymer in glass capillary.*

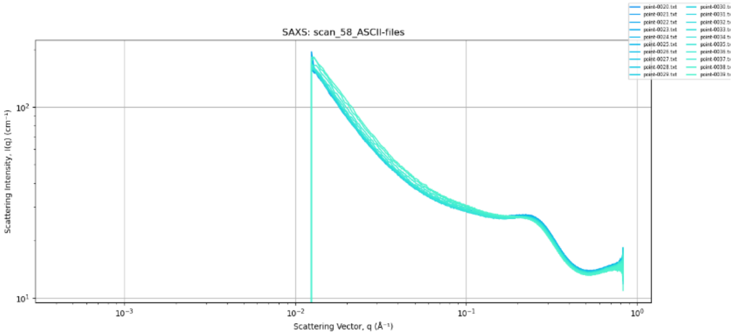

Figure S22. *Raw 1D plot for AMPS:PEGDA 30:70, 40 wt% total polymer in a glass capillary.*

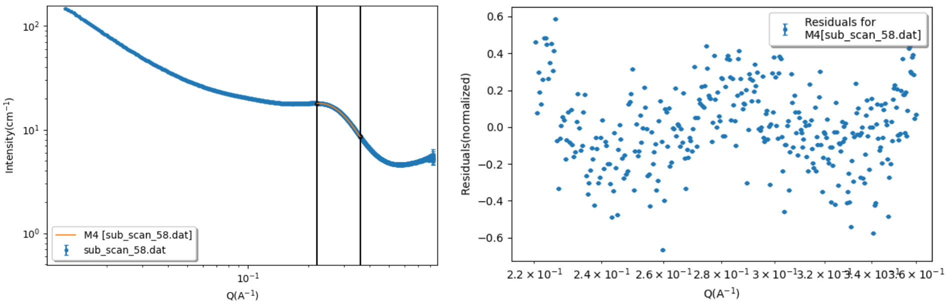

Figure S23. *Fitting of the background-subtracted 1D plot using the Teubner-Strey model in SasView 6.0 with a Q-range of 0.22-0.36 Å (left) and the corresponding residuals (right). Sample: AMPS:PEGDA 30:70, 40 wt% total polymer in glass capillary.*

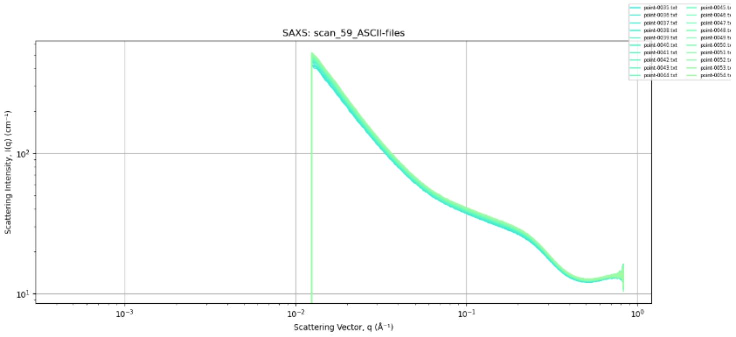

Figure S24. *Raw 1D plot for AMPS:PEGDA 30:70, 30 wt% total polymer in a glass capillary.*

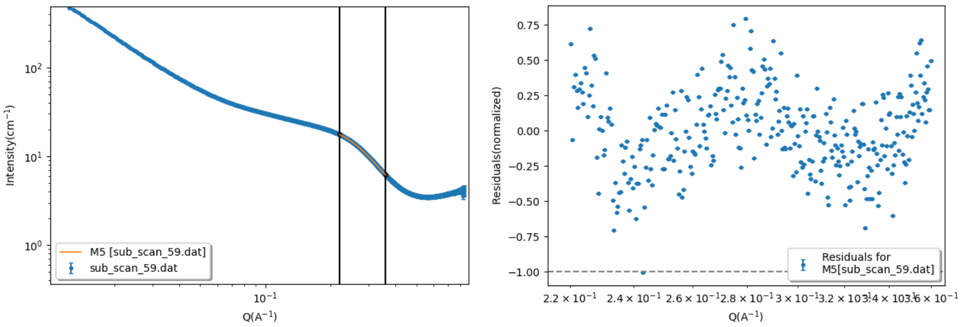

Figure S25 *Fitting of the background-subtracted 1D plot using the Teubner-Strey model in SasView 6.0 with a Q-range of 0.22-0.36 Å (left) and the corresponding residuals (right). Sample: AMPS:PEGDA 30:70, 30 wt% total polymer in glass capillary.*

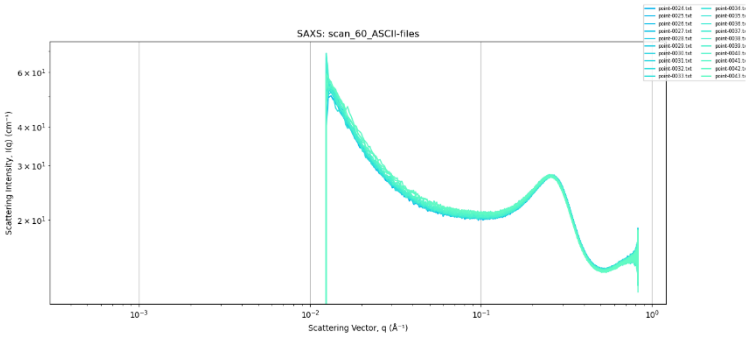

Figure S26. *Raw 1D plot for AMPS:PEGDA 50:50, 40 wt% total polymer in a glass capillary.*

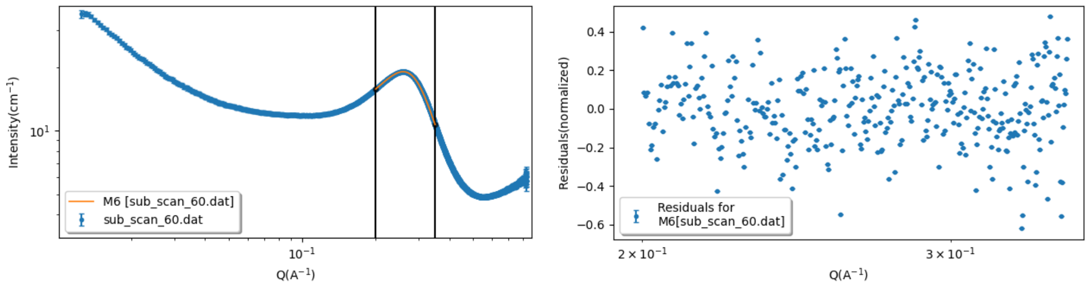

Figure S27. *Fitting of the background-subtracted 1D plot using the Teubner-Strey model in SasView 6.0 with a Q-range of 0.20-0.35 Å (left) and the corresponding residuals (right). Sample: AMPS:PEGDA 50:50, 40 wt% total polymer in glass capillary.*

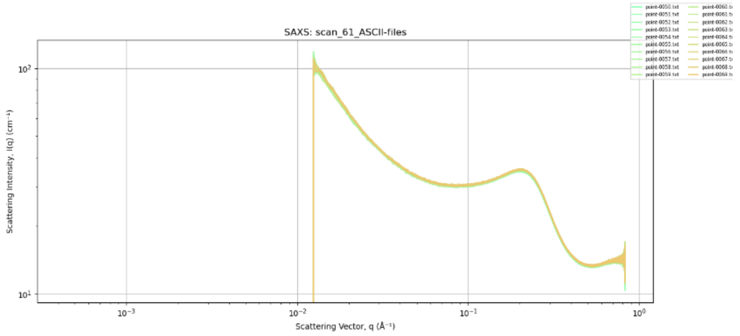

Figure S28. *Raw 1D plot for AMPS:PEGDA 50:50, 30 wt% total polymer in a glass capillary.*

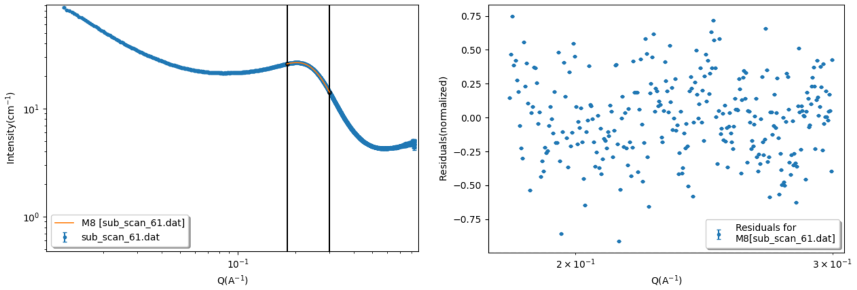

Figure S29. *Fitting of the background-subtracted 1D plot using the Teubner-Strey model in SasView 6.0 with a Q-range of 0.18-0.30 Å (left) and the corresponding residuals (right). Sample: AMPS:PEGDA 50:50, 30 wt% total polymer in glass capillary.*

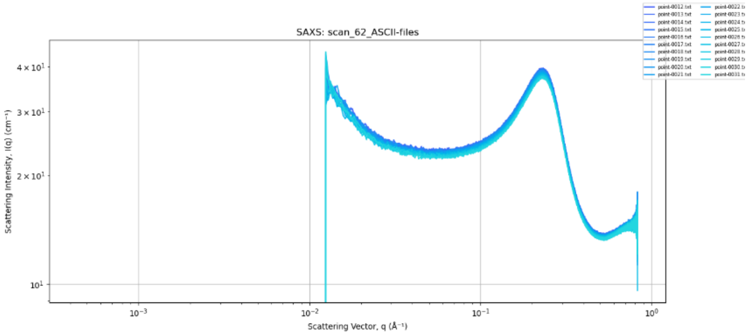

Figure S30. *Raw 1D plot for AMPS:PEGDA 70:30, 30 wt% total polymer in a glass capillary.*

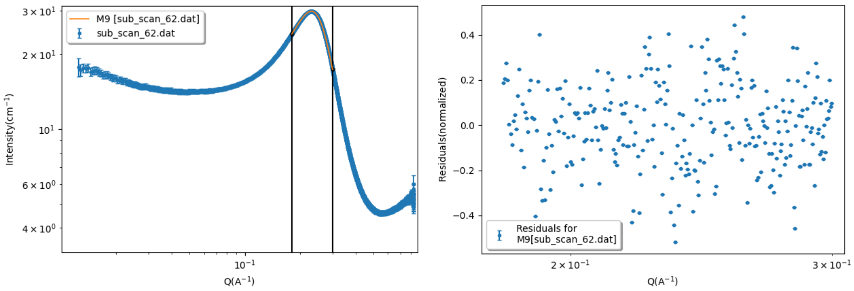

Figure S31. *Fitting of the background-subtracted 1D plot using the Teubner-Strey model in SasView 6.0 with a Q-range of 0.18-0.30 Å (left) and the corresponding residuals (right). Sample: AMPS:PEGDA 70:30, 30 wt% total polymer in glass capillary.*

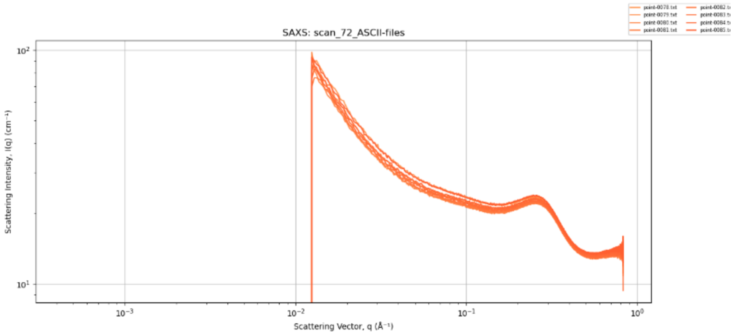

Figure S32. *Raw 1D plot for AMPS:PEGDA 30:70, 50 wt% total polymer in a free-standing sample.*

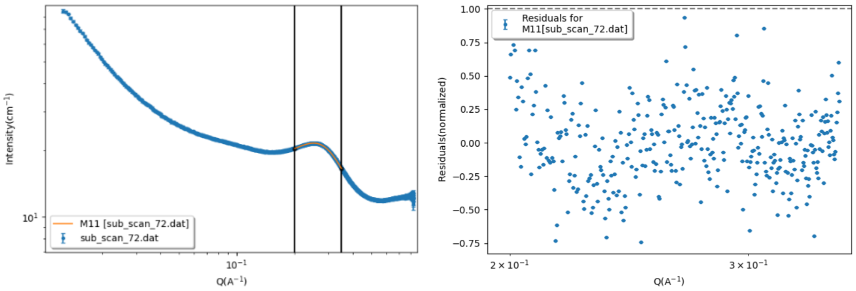

Figure S33. *Fitting of the background-subtracted 1D plot using the Teubner-Strey model in SasView 6.0 with a Q-range of 0.20-0.35 Å (left) and the corresponding residuals (right). Sample: Free-standing AMPS:PEGDA 30:70, 50 wt% total polymer.*

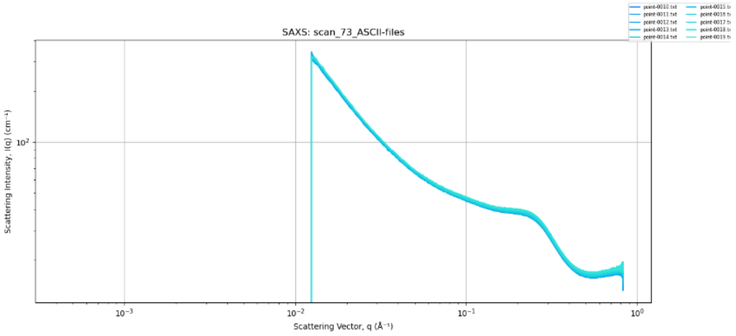

Figure S34. *Raw 1D plot for AMPS:PEGDA 30:70, 40 wt% total polymer in a free-standing sample.*

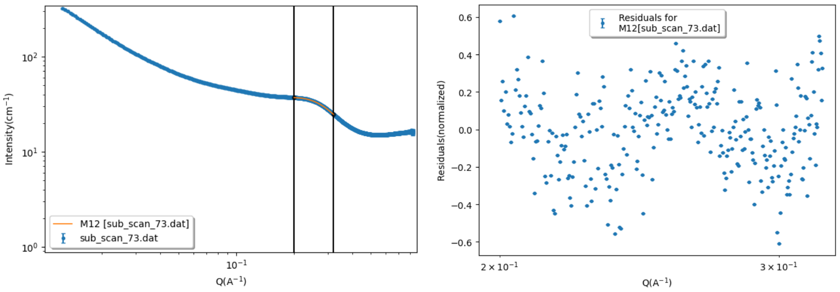

Figure S35. *Fitting of the background-subtracted 1D plot using the Teubner-Strey model in SasView 6.0 with a Q-range of 0.20-0.32 Å (left) and the corresponding residuals (right). Sample: Free-standing AMPS:PEGDA 30:70, 40 wt% total polymer.*

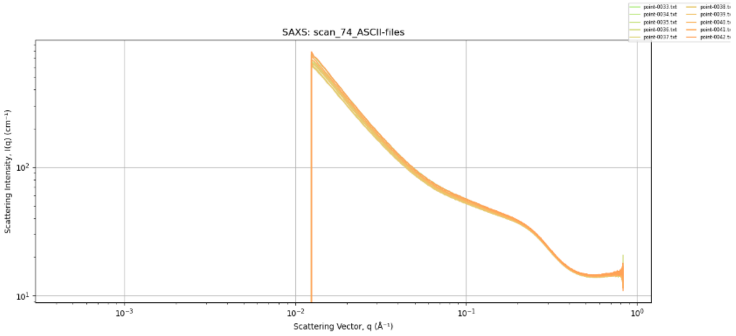

Figure S36. *Raw 1D plot for AMPS:PEGDA 30:70, 30 wt% total polymer in a free-standing sample.*

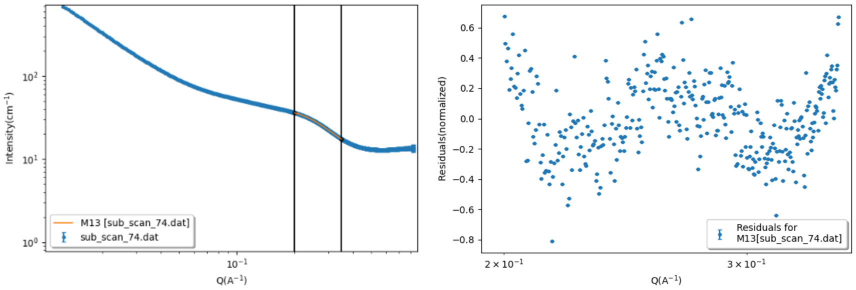

Figure S37. *Fitting of the background-subtracted 1D plot using the Teubner-Strey model in SasView 6.0 with a Q-range of 0.20-0.35 Å (left) and the corresponding residuals (right). Sample: Free-standing AMPS:PEGDA 30:70, 30 wt% total polymer.*

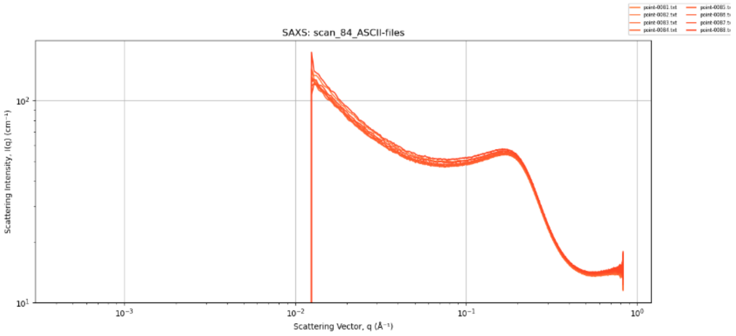

Figure S38. *Raw 1D plot for AMPS:PEGDA 70:30, 30 wt% total polymer in a free-standing sample.*

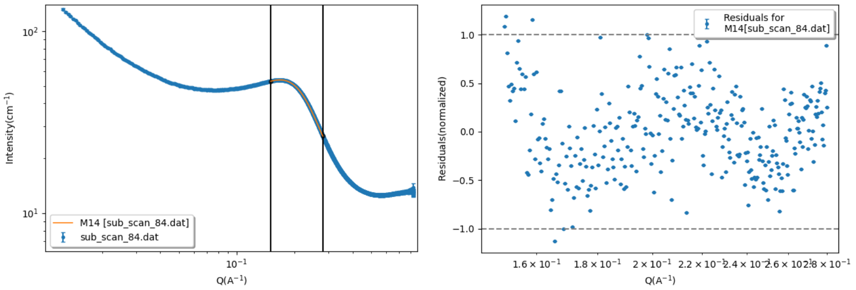

Figure S39. *Fitting of the background-subtracted 1D plot using the Teubner-Strey model in SasView 6.0 with a Q-range of 0.20-0.35 Å (left) and the corresponding residuals (right). Sample: Free-standing AMPS:PEGDA 70:30, 30 wt% total polymer.*

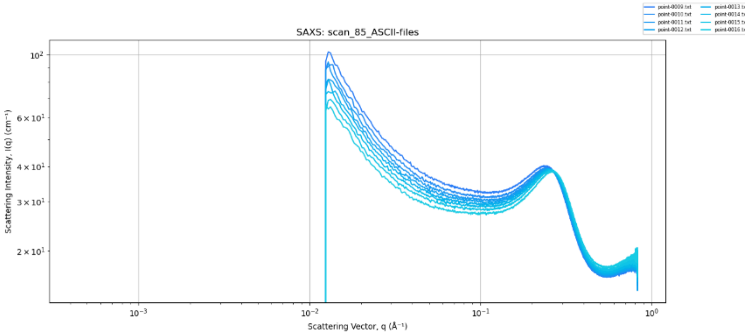

Figure S40. *Raw 1D plot for AMPS:PEGDA 50:50, 40 wt% total polymer in a free-standing sample.*

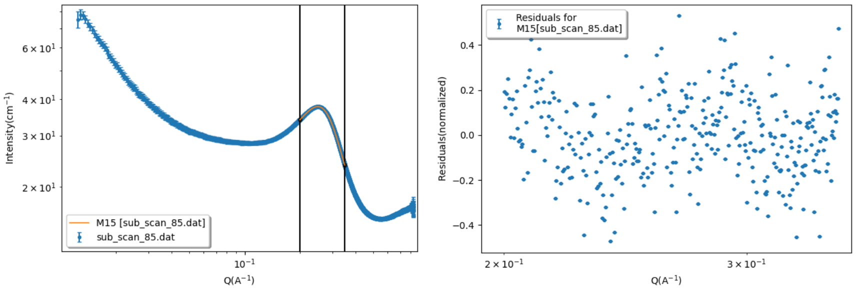

Figure S41. *Fitting of the background-subtracted 1D plot using the Teubner-Strey model in SasView 6.0 with a Q-range of 0.20-0.35 Å (left) and the corresponding residuals (right). Sample: Free-standing AMPS:PEGDA 50:50, 40 wt% total polymer.*

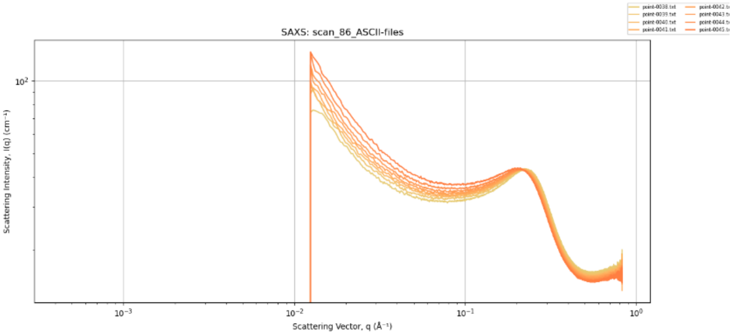

Figure S42. *Raw 1D plot for AMPS:PEGDA 50:50, 30 wt% total polymer in a free-standing sample.*

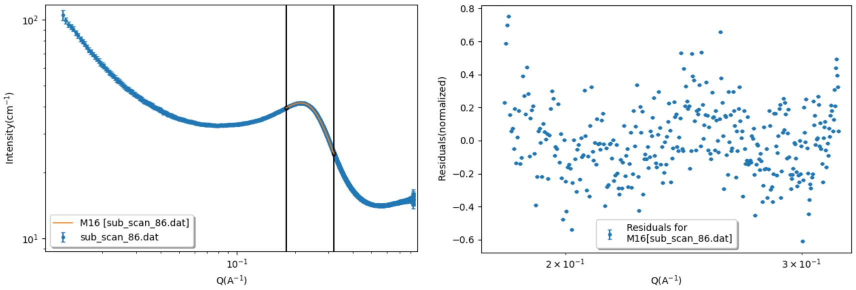

Figure S43. *Fitting of the background-subtracted 1D plot using the Teubner-Strey model in SasView 6.0 with a Q-range of 0.18-0.32 Å (left) and the corresponding residuals (right). Sample: Free-standing AMPS:PEGDA 50:50, 30 wt% total polymer.*

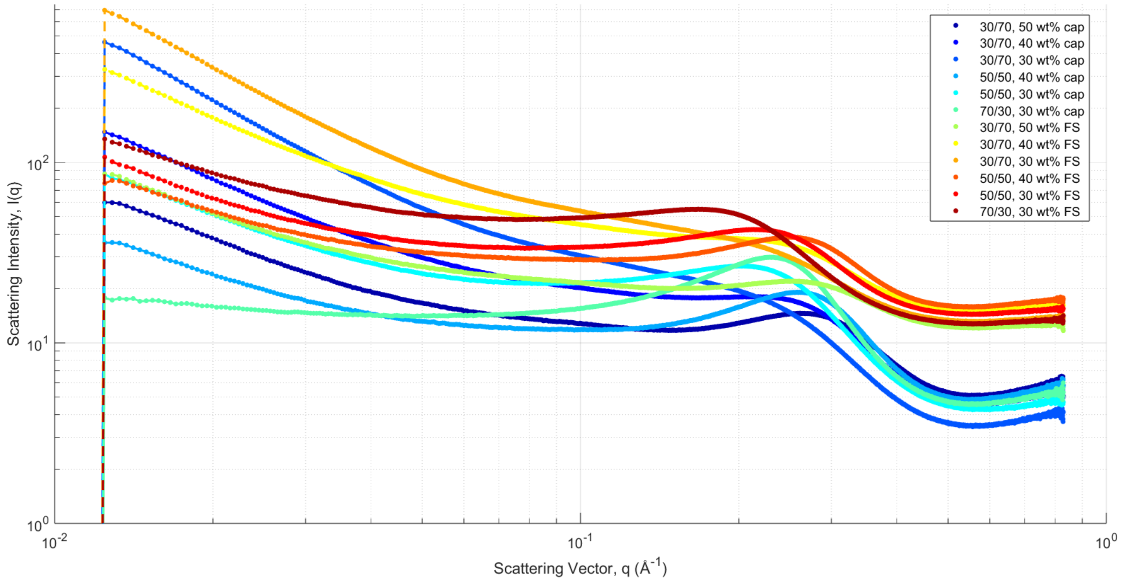

Figure S44. *Background-subtracted 1D SAXS profiles for both capillary and free-standing samples.*

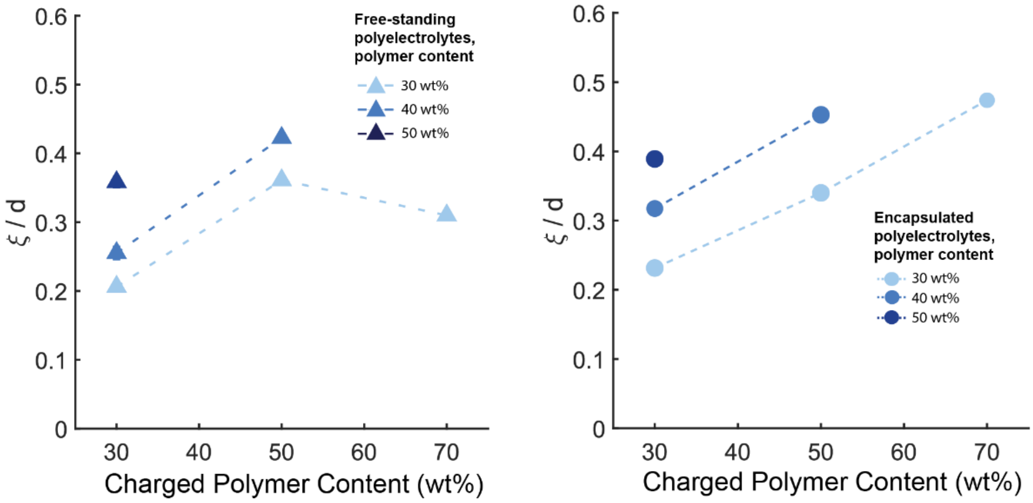

Figure S45. *Polydispersity, calculated by dividing the structural order parameter by the domain spacing (ξ/d) for both free-standing and encapsulated electrolytes.*

9. Delivery Rate Determination

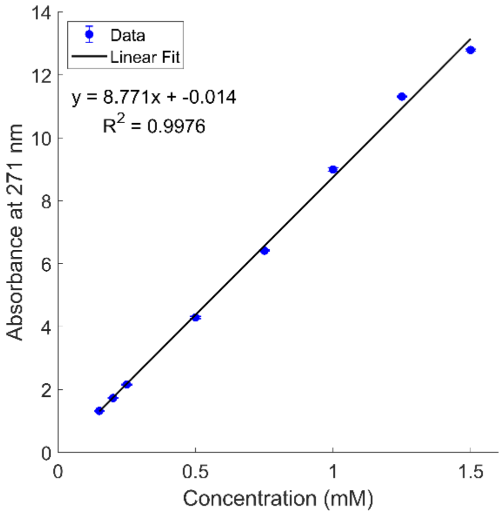

Figure S46. *Calibration curve for cytidine measured by UV–Vis absorbance at 271 nm. Data points represent mean values ± standard deviation (n = 3). The solid line indicates a linear least-squares fit.*

The delivery rate was calculated using the following equation:

$$Delivery\ rate\ [mol/s] = \frac{c_{cyt}\left[\frac{mol}{L}\right] \times V_{target}[L]}{time\ of\ delivery\ [s]}$$

The delivery efficiency was defined as the ratio between the experimentally delivered amount of cytidine ($n_{cyt} = c_{cyt} \times V_{target}$) and the theoretical maximum based on the applied current:

$$Efficiency = \frac{n_{\text{delivered}}}{I \cdot t/(zF)}$$

where I is the applied current, t is the delivery time, $z = +1$ is the charge of cytidine under the operating conditions, and $F = 9.6485 \times 10^4$ C mol^{-1} is Faraday's constant. In this study, the operating current was set to 50 nA, corresponding to a theoretical maximum of 31.1 pmol min^{-1}.

References

1. Yan, N., Paul, D. R. & Freeman, B. D. Water and ion sorption in a series of cross-linked AMPS/PEGDA hydrogel membranes. *Polymer* **146**, 196–208 (2018).

2. Yan, N. *et al.* Influence of fixed charge concentration and water uptake on ion sorption in AMPS/PEGDA membranes. *Journal of Membrane Science* **644**, 120171 (2022).

3. Jia, M., Luo, L. & Rolandi, M. Correlating Ionic Conductivity and Microstructure in Polyelectrolyte Hydrogels for Bioelectronic Devices. *Macromol. Rapid Commun.* **43**, 2100687 (2022).

4. Teubner, M. & Strey, R. Origin of the scattering peak in microemulsions. *The Journal of Chemical Physics* **87**, 3195–3200 (1987).

5. Hickey, R. J., Gillard, T. M., Irwin, M. T., Lodge, T. P. & Bates, F. S. Structure, viscoelasticity, and interfacial dynamics of a model polymeric bicontinuous microemulsion. *Soft Matter* **12**, 53–66 (2015).
